# A Hybrid Experimental and in silico Platform for ITPK1 Chemical Probe Discovery

**DOI:** 10.1016/j.slasd.2026.100323

**Published:** 2026-06-23

**Authors:** Adam Yasgar, Sankalp Jain, Huanchen Wang, Chih-Shia Lee, Guangning Zong, Haibo Zhang, Eric Lindberg, Carolyn Woodroofe, Kelly Lane, Burchelle Blackman, Dan Crooks, Hsiuling Lin, Bolormaa Baljinnyam, Michael Ronzetti, Anton Simeonov, Sandeep Rana, Ganesha Rai, Stephen Shears, Robin E. Stanley, Alexey V. Zakharov, Ji Luo, Natalia J. Martinez

**Affiliations:** a National Center for Advancing Translational Sciences, National Institutes of Health, Rockville, MD, USA; b Molecular and Cellular Biology Laboratory, National Institute of Environmental Health Sciences, National Institutes of Health, Research Triangle Park, NC, USA; c Laboratory of Cancer Biology and Genetics, Center for Cancer Research, National Cancer Institute, National Institutes of Health Bethesda, MD, 20892, USA; d Chemistry and Synthesis Center, National Heart, Lung and Blood Institute, Bethesda, MD, 20814, USA

**Keywords:** Chemical probes, High-throughput screening (HTS), ITPK1, PPIP5K2, Machine learning (ML), Quantitative structure activity relationship (QSAR)

## Abstract

Inositol-tetrakisphosphate 1-kinase (ITPK1) is a pivotal enzyme in the inositol phosphate signaling pathway that functions to maintain the balance of inositol phosphate (IP) species. Dysregulation of this pathway has been linked to human disease, making ITPK1 an attractive therapeutic target. While high-throughput screening (HTS) is a traditional strategy for identifying small molecule inhibitors, integrating computational approaches can significantly speed up and enhance hit rates. Here, we developed a hybrid experimental and virtual approach towards the identification of ITPK1 chemical probe candidates. We first miniaturized an ITPK1 enzymatic assay to 1536-well format and screened ~19,000 annotated and chemically diverse compounds. We then utilized the resulting dataset to develop Machine Learning (ML) and Pharmacophore (PH4) models to virtually screen a larger library of 120,000 compounds to expand the chemical diversity of the screening set. Importantly, our screening platform included a selectivity assay against PPIP5K2, the closest structural relative of ITPK1. The identified hits were evaluated for ITPK1 binding, including via a novel high-throughput Structure Dynamic Response (SDR) target engagement assay. Hits underwent further confirmation through orthogonal assays and mechanistic investigation, including obtaining a co-crystal structure for one of the hits. This integrated workflow—combining physical HTS with computational modeling—led to the identification of two novel candidate inhibitors. This study demonstrates an efficient, scalable strategy for targeting ITPK1 and offers a promising platform for drug discovery efforts in diseases linked to perturbed inositol phosphate pathways.

## 1. Introduction

Inositol tetrakisphosphate 1-kinase (ITPK1) is a key metabolic enzyme that regulates cellular signaling by balancing the synthesis and interconversion of inositol phosphate (IP) molecules. Specifically, ITPK1 phosphorylates inositol-1,3,4-trisphosphate [Ins(1,3,4)P_3_] and inositol-3,4,5,6-tetrakisphosphate [Ins(3,4,5,6)P_4_], generating inositol-1,3,4,5,6-pentakisphosphate (IP_5_), which can be further phosphorylated to become inositol hexakisphosphate (IP_6_). ITPK1 also has phosphatase activity, converting IP_5_ back to Ins(3,4,5,6)P_4_ [1]. These IP molecules are key signaling metabolites that influence calcium homeostasis, cytoskeletal dynamics, vesicle trafficking, and gene expression [2]. Through these functions, ITPK1 helps coordinate cellular responses to growth factors and environmental cues. Dysregulation of ITPK1 activity has been implicated in multiple disease contexts, including cancer, metabolic disorders, and neurological conditions, where altered inositol phosphate signaling can disrupt cell proliferation, migration, and survival pathways [3]. Altogether, these functions position ITPK1 as a potential therapeutic target in diseases driven by aberrant inositol signaling [4].

**Fig. 1.**
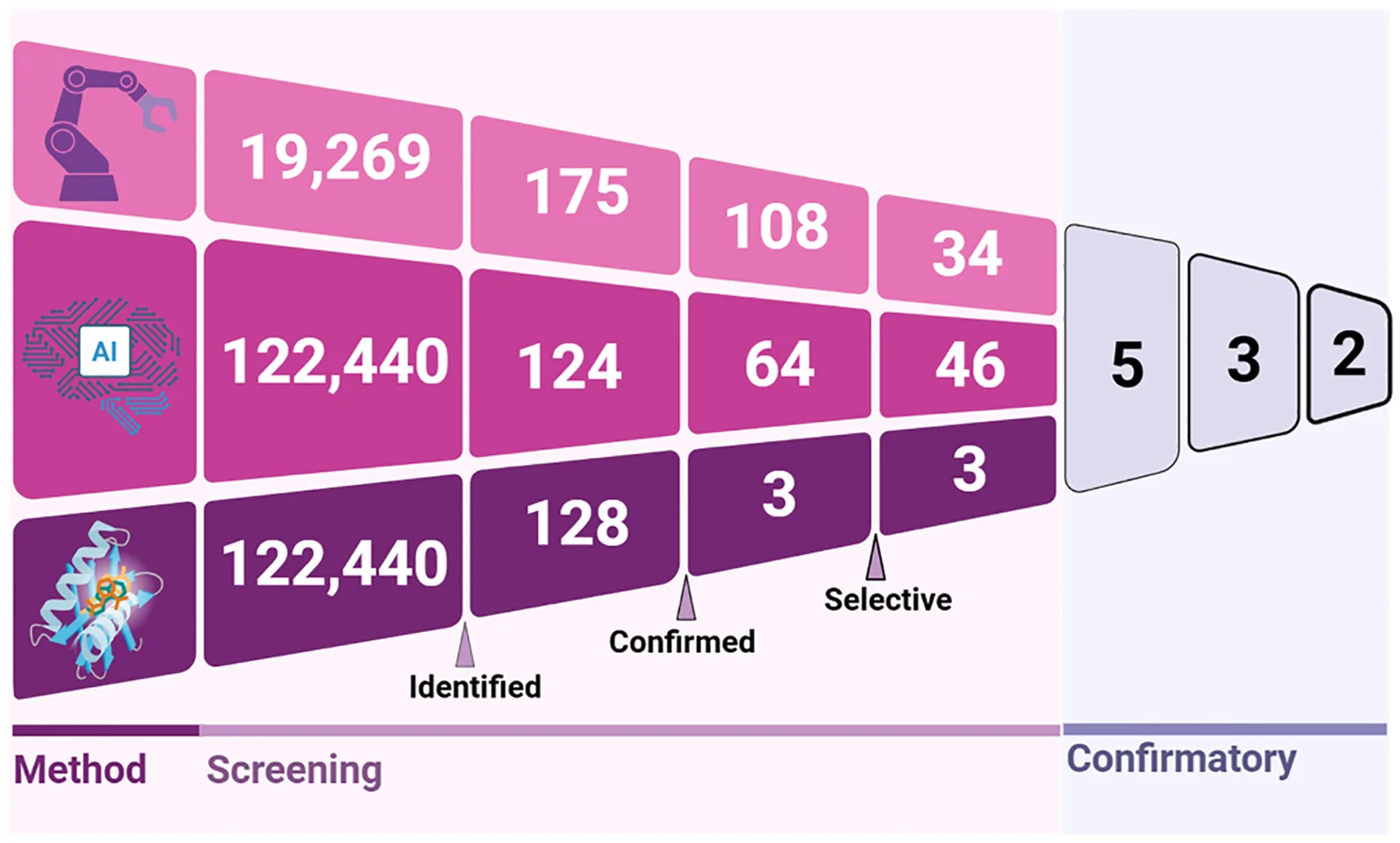
Schematic representation of screening strategy. Combined approach of qHTS (traditional) and virtual screening (machine learning and docking, middle and bottom rows, respectively) leading to the identification of candidate inhibitors for ITPK1. Each step lists the number of compounds screened, tested, confirmed, and selective using each screening method.

The first potent inhibitor (IC_50_ <100 nM) of ITPK1, 9-cyclopentyladenine (9-CPA), was identified from a high-throughput screen of 54,000 compounds using a 384-well ITPK1 enzymatic assay. Using a variety of techniques, 9-CPA was shown to not only bind to purified ITPK1 but also inhibit its function in cells, disrupting IP_5_ and IP_6_ levels [5]. While a remarkable accomplishment, 9-CPA lacks a key feature typically required of a chemical probe or lead compound, specifically, potent cellular activity [6]. In this study, we established a comprehensive platform to facilitate the discovery of novel chemical probes for ITPK1. We employed a hybrid strategy combining experimental and virtual approaches to rapidly identify ITPK1 inhibitors. We first miniaturized an ITPK1 ADP-Glo^™^ assay to a high-density 1536-well format (4 μL volume), enabling quantitative high-throughput screening (qHTS), a superior screening approach shown to minimize false negative and false positive rates [7–9]. Following a pilot screen, we utilized machine learning (ML) and pharmacophore (PH4) models to virtually screen 120, 000 compounds. To ensure specificity, the platform incorporated high-throughput selectivity screening against PPIP5K2 (the closest structural relative of ITPK1) alongside high- and medium-throughput target engagement assays. A co-crystal structure of a novel hit was obtained to guide future optimization efforts. This approach, depicted in Fig. 1, resulted in the identification and validation of two small molecule inhibitors with significant potential for probe development.

## 2. Results

### 2.1. Optimization of ITPK1-ADP-Glo^™^ assay in 1536-well format

To identify ITPK1 small molecule inhibitors, we sought to implement a 1536-well based assay to support qHTS. We were guided by the 384-well Kinase-Glo luminescence assay reported by Ng et al., in which a coupled luciferase assay was used to quantify phosphatase ITPK1 activity using ADP and IP_5_ substrates [5,10]. To speed up the reaction and achieve greater sensitivity in 1536-well format, we changed the detection readout to the homogeneous ADP-Glo luminescence assay [11]. This allowed us to monitor ADP production by driving the reaction in the kinase direction, phosphorylating the Ins(3,4,5,6)P_4_ substrate to IP_5_ product. We implemented the same assay development on PPIP5K2, a kinase responsible for the generation of inositol pyrophosphates IP_7_ and IP_8_, which was chosen for selectivity as it is the closest structural relative to ITPK1 [12,13]. For this enzyme, we used 5-IP_7_ as the substrate.

We first performed enzyme titrations for both ITPK1 and PPIP5K2 ranging from 25 nM to 200 nM in the presence of 1 μM Ins(3,4,5,6)P_4_ substrate for ITPK1 and 1 μM 5-IP_7_ substrate for PPIP5K2, respectively, with 10 μM ATP for both proteins (see Methods). Substrate concentrations were kept well above K_m_ while the cofactor ATP was maintained at K_m_ (a difference from previous methods [12]) enabling us to preferentially identify substrate competitive inhibitors. Luminescence was assessed at 3, 5, 15, 30, 60, 120, 180 and 240 min (Figs S1A–B). Balancing enzyme amount and incubation time, we chose a starting concentration of 100 nM of each enzyme at 60 min (~88% and 73% conversion for ITPK1 and PPIP5K2, respectively) for testing to allow for optimal assay conditions (Figs S1C–F) [13,14]. The Z’-factor (Z’) was calculated from the change in luminescence intensity over the respective reaction period and normalized against no-inhibitor or no-enzyme for neutral (DMSO) or positive controls, respectively. At ≥60 min, the signal to background (S:B) value was relatively stable (>10) at ≥100 nM ITPK1, with Z’ values >0.8, indicating acceptable assay performance.

### 2.2. Quantitative high-throughput screening: pilot screen

**Fig. 2.**
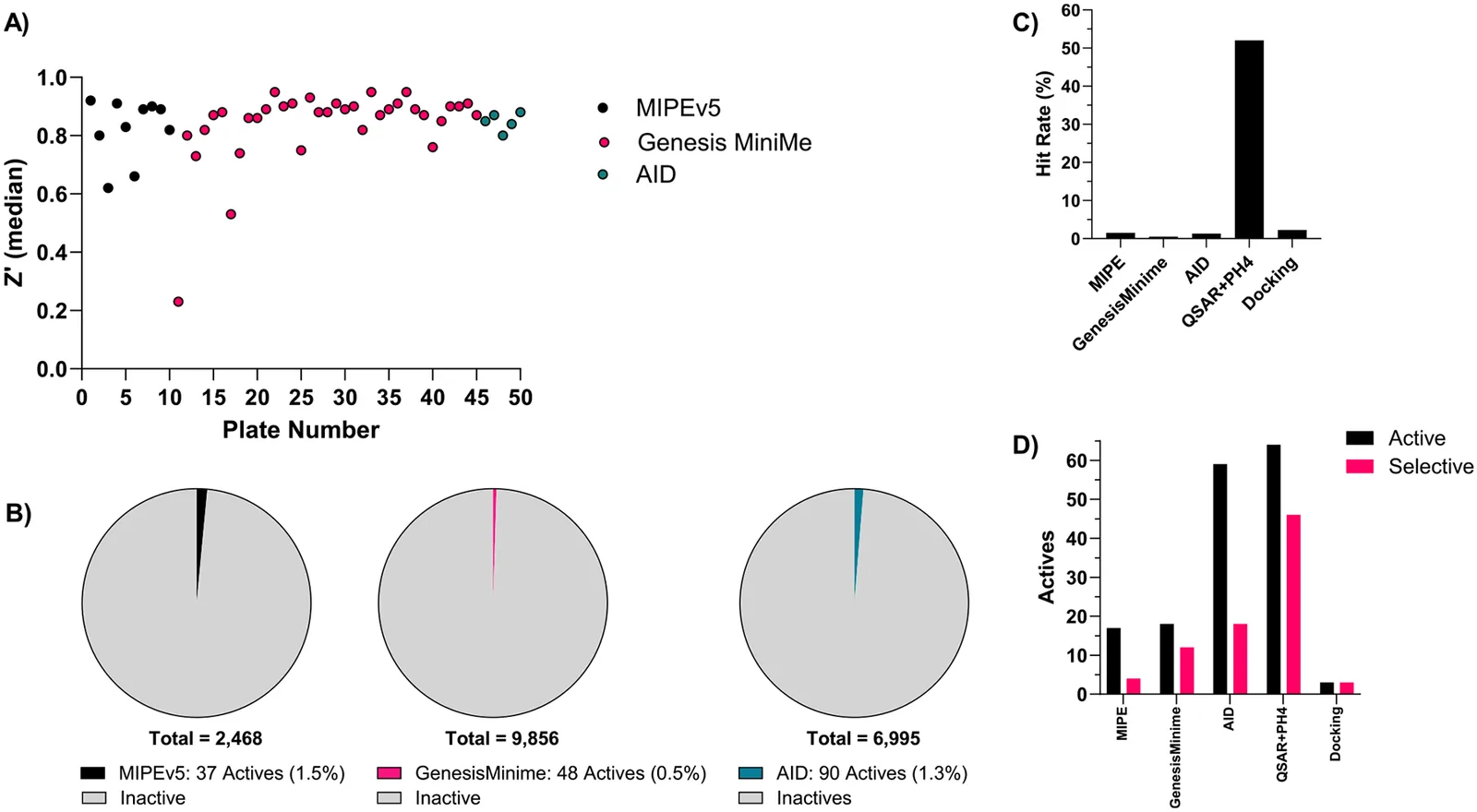
In vitro and in silico screening performance and selectivity landscape of hits. (A) Assay performance (Z’-factor) across the screening libraries. (B, C) Hit rates and activity profiles of compounds identified through traditional high-throughput screening and virtual screening campaigns. (D) Selectivity assessment of confirmed ITPK1 hits evaluated against PPIP5K2.

We tested the previously assembled MIPE 5.0 [15] compound library of >2400 oncology-related compounds in the miniaturized ADP-Glo assay against ITPK1. Notably, this library includes 603 kinase inhibitors. Compounds were tested at 5 concentrations (final concentration range of 2 nM to 100 μM) to generate concentration-response curves (CRC), enabling us to prioritize active samples [7,16,17]. The assay performance of the screen was excellent, with a cumulative Z’ of 0.83 ± 0.11 and S:B of 15.3 ± 3.2 (Fig. 2A).

We identified 37 compounds with high quality dose response curves (1.5%; see Methods for criteria) as potential ITPK1 inhibitors (Table S1, 2B and 2C). We then retested 36 available compounds in high-density dose-response curves (11-points, 1:2 dilution, for a final concentration of 100 nM to 100 μM) against both the ITPK1 and PPIP5K2 assays for selectivity assessment. We also included a counterscreen assay to eliminate any compound interfering with the ADP-Glo readout (Tables S2 and S3, respectively). Of the 36, 17 (47%) were confirmed as inhibitors, exhibiting moderate to weak inhibitory potency against ITPK1 with an IC_50_ range of 9.3 to 39.8 μM. Of the 17, 13 (77%) were also active against PPIP5K2, leaving four apparent selective (defined as an IC_50_ ratio >5) candidate inhibitors (Fig. 2D). While it is generally recommended to have >30-fold selectivity within a target family for an optimized chemical probe, given the low hit rate we chose a value of >5-fold for the initial screening hits [18]. None of the selective hits interfered with the assay readout (Table S2). Three of the selective compounds were weak (IC_50_ >30 μM and/or < −50% efficacy; Table S1), with only one compound, Idronoxil (NCGC00346822), displaying an IC_50_ of 9.3 μM vs. >50 μM for ITPK1 and PPIP5K2, respectively (Fig. S2A). For reference, 9-cyclopentyladenine (9-CPA, NCGC00162114, PubChem CID 12,852) displayed IC_50_ values of 0.092 μM and >50 μM vs. ITPK1 and PPIP5K2, respectively (Fig. S2B), in agreement with the previously published result.

Our confirmatory screen yielded no active hits among the 603 kinase inhibitors tested. These findings, consistent with those of Ng et al., suggest that the pharmacology of ITPK1 requires chemical diversity not previously screened. While the compounds identified in the pilot screen were not as potent as 9-CPA, they served as a good indicator of how the screen might perform when scaled up to larger compound libraries.

### 2.3. Quantitative high-throughput screening: medicinal chemistry compound libraries

To identify novel chemical matter for ITPK1 we screened internal compound collections curated for synthetic tractability and structural diversity, ensuring the identified hits would be amenable to downstream optimization [19]. First, we screened a subset of the Genesis Library (GenesisMinime) which contains representative scaffolds that can be used to identify starting points for medicinal chemistry programs [20,21]. A total of 9856 compounds were screened in qHTS format (see Methods). We observed an excellent mean Z’-factor of 0.84 ± 0.13 (Fig. 2A). A combination of low hit rates and weak actives were observed, with 48 (0.5% hit rate) compounds exhibiting inhibition (Table S4 and Figs. 1, 2B, and C). Upon retesting in high-density dose response curves, 18 out of 48 (38%) confirmed, with 12 selective for ITPK1 (Fig. 2D). Most of the selective compounds were weak (IC_50_ >30 μM and/or >−50% efficacy), with only one compound (NCGC00527096) displaying an IC_50_ < 30 μM and efficacy < −50% (Fig. S2C).

Next, we screened our internally designed Artificial Intelligence Driven (AID) Library of 6995 compounds, a subset selected from the Enamine Targeted Library [22]. The AID Library offers wider chemical diversity and opportunity for additional chemical matter. Due to resource limitations, this library was tested against ITPK1 at a single concentration (50 μM final concentration) using our fully automated robotic system (Table S5; see Methods) [23]. Again, we observed excellent assay performance across the five-plates tested, with a mean Z’-factor of 0.85 ± 0.03 (Fig. 2A). We utilized a modified triage strategy because of the single-concentration experiment, using an efficacy cutoff of 50% to identify candidate inhibitors for confirmatory testing in dose-response. With those criteria, we identified 90 compounds (1.3% hit rate) for further testing in dose-response (Fig. 2B). 59 compounds confirmed, of which 18 (30% of actives) were selective towards ITPK1 versus PPIP5K2 (Figs. 1, 2D and Table S6). Again, most of the selective compounds were weak (IC_50_ >30 μM and/or >−50% efficacy), with only one compound (NCGC00879727) displaying an IC_50_ < 30 μM and efficacy < −50% (Fig. S2D).

### 2.4. Virtual screen: machine learning and docking/structure-based modeling

To expand chemical diversity and improve the efficiency of hit identification for ITPK1 inhibition, we implemented an in silico screening strategy combining Quantitative Structure Activity Relationship (QSAR) modeling with ligand-based pharmacophore (PH4) approaches. These computational approaches have been increasingly recognized as efficient and productive tools for identifying lead candidates within the chemical probe discovery process, offering improved hit rates, enhanced scaffold diversity, and reduced experimental burden [22,24,25]. However, a high-quality training set is essential for these models to be successful. While the initial HTS campaigns (Fig. 1) produced primarily weak, albeit selective, ITPK1 inhibitors, they provided a well-annotated set of confirmed active and inactive suitable for model development.

We developed a deep learning consensus architecture (DLCA) classification model using the activity data derived from a curated subset of the HTS campaign (see Methods). The model showed reasonable external validation performance (Precision = 0.64; AUC = 0.74) and was then applied to the full Genesis collection of 120,000 compounds [20], resulting in 1266 compounds predicted to be active against ITPK1 (Table S7; see Methods). In parallel, ligand-based pharmacophore models were generated from the most potent and structurally representative active compounds (Table S8; see Methods). Compounds ranking highly in both the DLCA predictions and pharmacophore fit scores were prioritized. From this overlap, 124 compounds were selected for biochemical evaluation against ITPK1, followed by PPIP5K2 to assess selectivity (Table S9). Out of these, 64 (52%) were active against ITPK1, and an impressive 46 (37%) showed selectivity over PPIP5K2 (Fig. 1 and Fig. 2C–D; none were active in the counterscreen). Relative to our combined traditional HTS screening hit rate of 0.9%, the consensus in silico approach produced a ~58-fold enrichment in confirmed actives. We then evaluated potency as a separate filter on hit quality. Despite the higher hit rate, only three compounds met our potency criteria (IC_50_ values < 30 μM and efficacy < −50%). Among these, only one compound (NCGC00418159) demonstrated clear selectivity for ITPK1 (Fig. S2E).

To complement the ligand-based models, we also performed structure-based docking using the crystal structure of ITPK1 (PDB ID: 2Q7D) against the entire Genesis collection (see Methods). This workflow yielded 128 compounds for experimental testing (Table S10). Of these, three showed measurable activity (2.3% hit rate; Fig. 2C and D), and just one (NCGC00410413) met potency criteria of IC_50_ < 30 μM and efficacy < −50% (Fig. S2F). The relatively low hit rate suggests that structure-based docking, as implemented here, was less effective than the ligand-based approaches for prioritizing ITPK1 inhibitors.

In total, 83 hits were identified through both empirical and computation screening. Based on potency, selectivity profile, and structure attributes amenable to medicinal chemistry optimization, we selected 4 of the compounds mentioned above (Fig. 1, S2A, S2C–F; Table S11). Additionally, we selected the relatively potent NCGC00418159 analog, NCGC00418121 (Fig. S2G), despite its lack of desired selectivity, totaling 5 compounds for follow up characterization. Lastly, NCGC00410413 was excluded from further evaluation due to loss of activity in the presence of 1 mM DTT, indicative of potential thiol reactivity.

### 2.5. Radiolabeled orthogonal assay

**Fig. 3.**
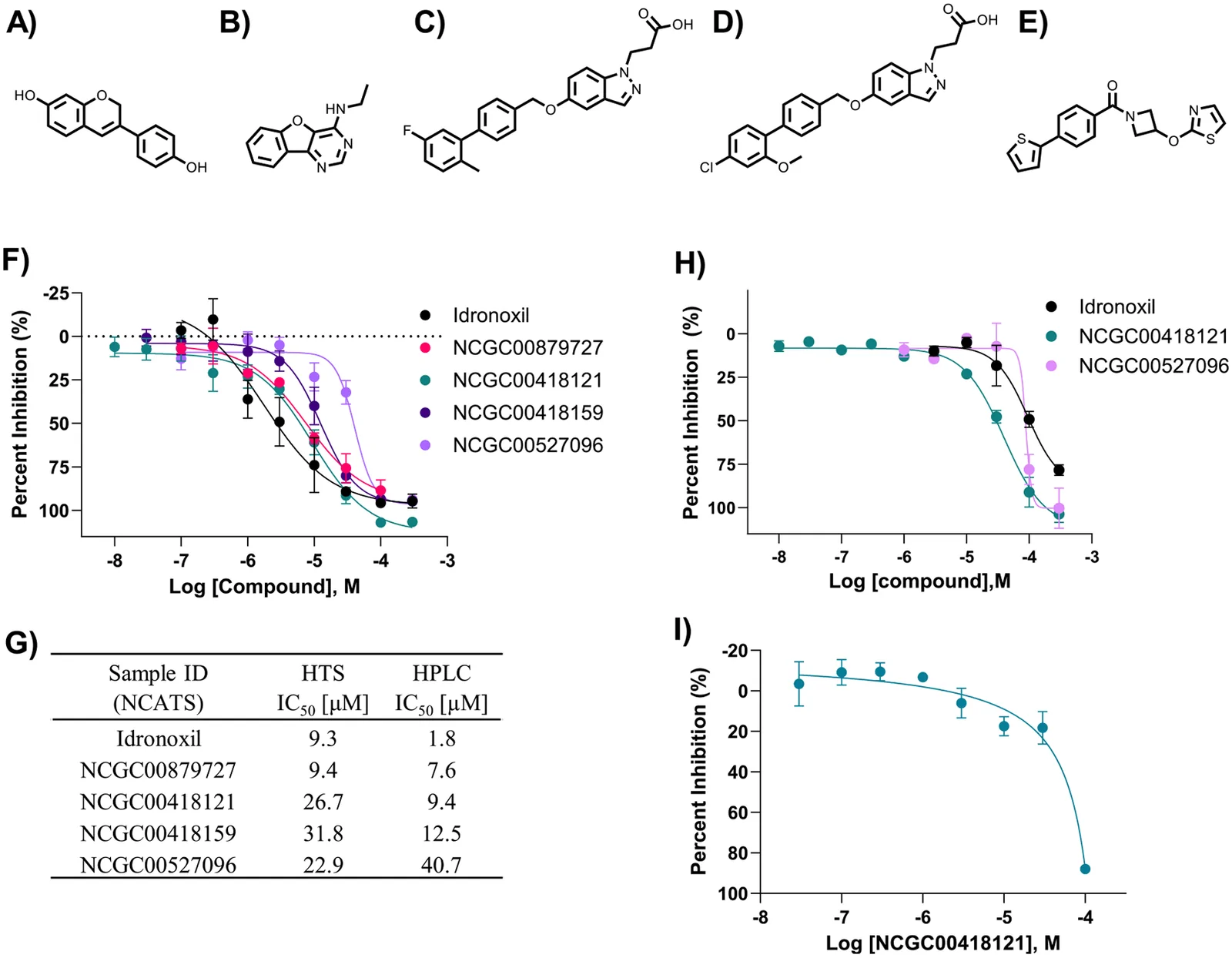
Radiolabeled orthogonal assay. (A – E) Chemical structures for Idronoxil, NCGC00879727, NCGC00418121, NCGC00418159, and NCGC00527096, respectively. (F) Dose-response curves of hits in radiolabeled assay (●)Idronoxil, (●)NCGC00879727, (●)NCGC00418121, (●)NCGC00418159, and (●) NCGC00527096 against ITPK1. (G) IC_50_ values [μM] of hits in the HTS and radiolabeled assays (H) Dose-response of candidate inhibitors against PPIP5K2 in radiolabeled assay. (I) Dose-response testing of (●)NCGC00418121 against the upstream inositol polyphosphate multikinase (IPMK) in radiolabeled assay.

To validate the compounds identified using the ADP-Glo readout, we employed the previously developed low-throughput radiometric IP kinase assay which utilizes HPLC to monitor metabolism of radiolabeled substrate [5,12]. Briefly, we tested the 5 compounds described above in dose-response (8- or 10-points; final concentration range of 100 nM to 300 μM) in a mixture containing 0.26 nM ITPK1, 10 μM ATP, and 1 μM Ins(3,4,5,6)P_4_ containing 100 K dpm ^33^P-Ins(3,4,5,6)P_4_, at 37 °C for 60 min in assay buffer to measure IP_5_ product formation (see Methods). All five candidate inhibitors confirmed, with IC_50_ values ranging from 1.8 to 40.7 μM (Fig. 3A, B, Table S11). Idronoxil and NCGC00879727 performed similarly to the HTS assay, with IC_50_ values of 1.8 μM and 7.6 μM, respectively. The modeling-derived compounds NCGC00418121 and NCGC00418159 exhibited improved potency compared to the HTS assay, with IC_50_ values of 9.4 μM and 12.5 μM, respectively, while NCGC00527096 proved to be the weakest, with an IC_50_ value of 40.7 μM (~2X weaker than the HTS luminescence assay). The HTS and radiolabeled assay-derived values were in line with those observed with the reference compound, 9-CPA, which was 0.092 μM and 0.052 μM in the enzymatic and radiolabeled assays, respectively [5].

The same set of compounds were then tested in the same radiometric orthogonal assay for selectivity using PPIP5K2 and ^33^P-5-IP_7_ substrate (see Methods). Compounds were first tested at a single concentration of 100 μM (Fig. S3A and Table S11), then tested in dose-response if inhibition was observed (Fig. 3C). The candidate inhibitors performed similarly to the PPIP5K2 HTS results, with three compounds exhibiting weak activity: Idronoxil, NCGC00418121, and NCGC00527096, with weak potencies of 94.8, 39.7, and 88.7 μM, respectively (Fig. 3C). Overall, the radiometric assay ranked compounds similarly to the HTS assay, in terms of potency and selectivity for ITPK1 and PPIP5K2.

Finally, we incorporated the previously studied inositol polyphosphate multikinase (IPMK) in the radiometric orthogonal assay with 1 μM [33P](1,3,4,5)P_3_ substrate (see Methods) for further assessment of selectivity [26]. IPMK functions upstream of ITPK1 in the inositol phosphate pathway, thereby enabling assessment of on-pathway selectivity. Compounds were tested first at a single concentration of 100 μM (Fig. S3B). Only NCGC00418121 exhibited single-point inhibition and was subsequently tested in dose-response, displaying a weak IC_50_ of ~50 μM (Fig. 3D). These results validate that the assay can be used as an additional tool to determine compound promiscuity. The potency of NCGC00527096 was similar for ITPK1 and PPIP5K2, hence this compound was filtered out (Table S11). Altogether, four of the five compounds remained selective towards ITPK1 and were selected for further characterization.

### 2.6. In vitro binding and target engagement studies

**Fig. 4.**
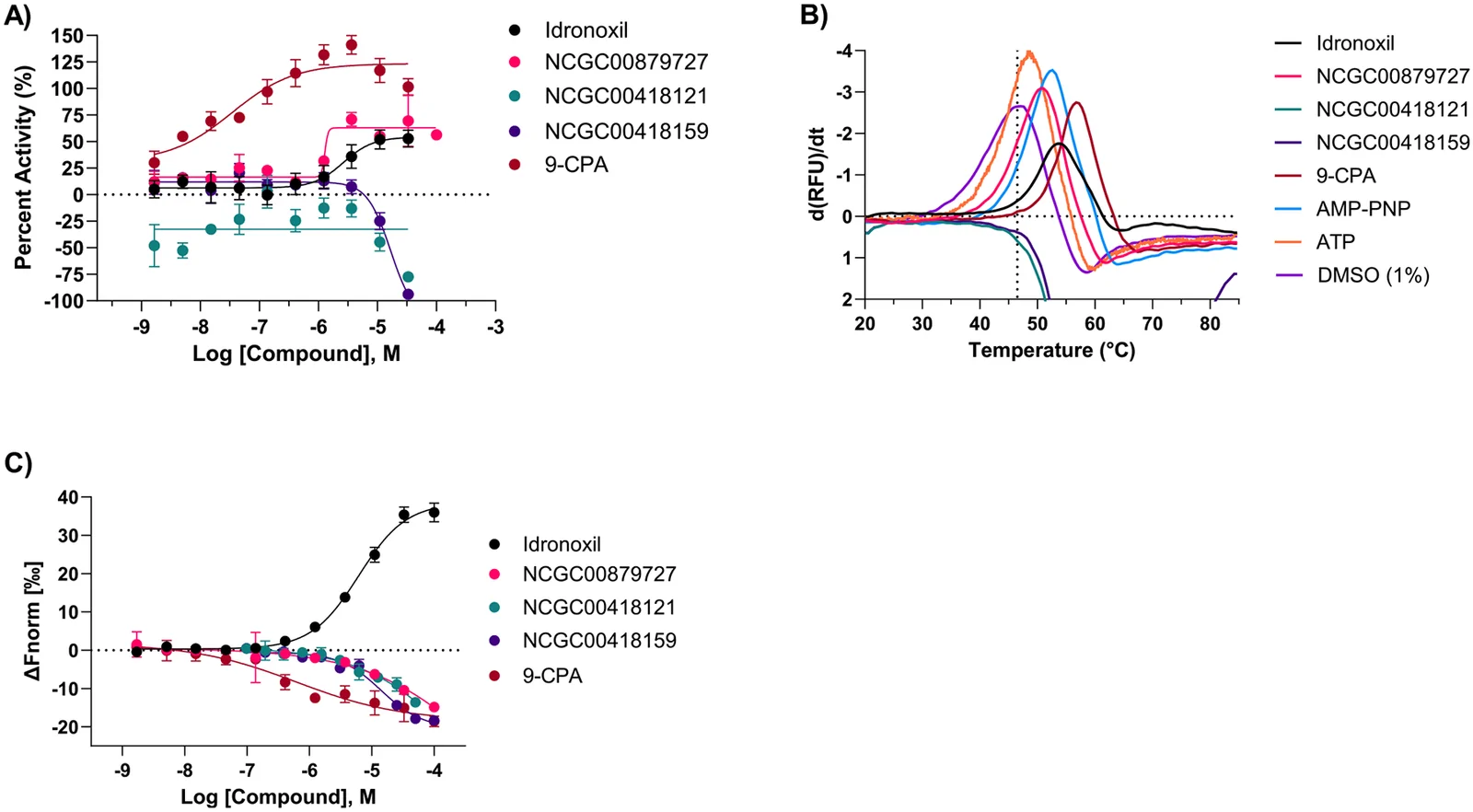
In vitro Binding and Target Engagement Studies. (A) Evaluation of candidate inhibitors in the Structural Dynamic Response (SDR) target engagement assay using cell lysates exogenously expressing HiBiT-ITPK1. (B) Representative Protein Thermal Shift Assay (TSA) melt profiles of candidate inhibitors [100 μM or 1% DMSO] assessing the thermal stabilization of ITPK1. (C) Representative Microscale Thermophoresis (MST) assay determining the binding affinity (K_d_) of compounds to fluorescently labeled ITPK1.

To complement the functional readouts provided by the above assays, we utilized orthogonal approaches to prove that the compounds are engaging with ITPK1 [27]. First, we evaluated the recently published ligand-dependent structural dynamics (SDR) assay, in which conformational changes induced by ligand binding to the target of interest can be transmitted to an NLuc sensor protein that is fused to the target [28]. Specifically, we developed a 1536-well assay to monitor compound-induced SDR of a reconstituted NLuc sensor, in which the large fragment of NLuc (LgBiT) is added to lysates of cells exogenously expressing ITPK1 that is fused to the small fragment of NLuc (HiBiT; see Methods and Fig. S4). Briefly, we tested the above compounds in dose-response (11-points; final concentration range of 1.7 nM to 99 μM) in cell lysates of HEK293T cells expressing HiBiT-ITPK1. To benchmark the assay, we tested 9-CPA, where we observed an increase in NLuc signal with an AC_50_ value of 29.5 nM (Fig. 4A and Table S11). Idronoxil and NCGC00879727 exhibited activity, with AC_50_ values of 4.16 and 1.00 μM, respectively, while NCGC00418121 was inactive/inconclusive and NCGC00418159 exhibited an AC_50_ value of 13.5 μM (Fig. 4A and Table S11). The other two compounds, NCGC00418121 and NCGC00418159, elicited a decrease in NLuc signal. We performed a counterscreen assay where no activity was observed, eliminating the possibility of compounds interfering with the NLuc readout (see Methods).

Next, we employed a medium-throughput Protein Thermal Shift Assay (TSA or Differential Scanning Fluorimetry, DSF) using SYPRO Orange, an environmentally sensitive hydrophobic dye, to determine the ability of ligands to thermally stabilize ITPK1 (see Methods) [29–31]. TSA provides a medium-throughput approach for identifying compound-target binding interactions using recombinant protein. At a single concentration point (100 μM), ATP co-factor and the inactive substrate mimetic AMP-PNP, induced a ΔT_m_ shift for ITPK1 of ~1.5 and ~5.5 °C, respectively, indicating this assay is suitable for interrogation of ITPK1-ligand interactions (Fig. 4B and Table S11; see Fig. S5A for additional concentration-melt profiles of AMP-PNP). The reference compound 9-CPA, Idronoxil, and NCGC00879727 produced a ΔT_m_ shift of ~10, 7, and 4 °C respectively, at 100 μM (Fig. 4B and Table S11). A dose-response TSA indicated that Idronoxil and NCGC00879727 elicit EC_50_ values of ~6 μM and ~100 μM, respectively (Fig’s S5C and S5D; for dose-response melting curves, raw signal, and counterscreen data see Fig’s S5E – S5P). We were unable to determine if stabilization was occurring for NCGC00418121 and NCGC00418159 as both exhibited high fluorescence (RFU) values in their melt profiles indicating the compounds have intrinsic fluorescence in the same range as SYPRO orange, leading to thermal profiles that are not reliable (Fig. 4B, Figs. S5K – S5P) [29].

Lastly, we adopted the previously published Microscale Thermophoresis (MST) assay [6] in combination with Spectral Shift [32] to leverage the platforms ability to monitor the binding interaction of a small molecule to a RED-Tris NTA-tagged protein [33,34] based on thermophoretic movement. Importantly, the RED-Tris NTA dye has a right-shifted fluorescent spectrum relative to SYPRO–Orange, which should mitigate the interference seen with NCGC00418121 and NCGC00418159. We tested 9-CPA and inhibitor candidates in dose-response (11-points; final concentration range of 1.7 nM to 100 μM) against fluorescently labeled ITPK1-(His)_6_ (see Methods). For 9-CPA we obtained a K_d_ of 0.367 μM (Fig. 4C and Fig. S6A), similar to the previously published value 0.11 μM [5]. We also observed binding for Idronoxil, NCGC00879727, and NCGC00418159, with K_d_ values of 6.2, 39.9, and 14.1 μM, respectively (Fig. 4C and Fig. S6B – D, respectively). For NCGC00418121, in the MST assay we observed inconclusive binding due to weak activity (Fig. 4C and Fig. S6E). We then switched to Spectral Shift, where we observed similar weak binding (Fig. S6F). The calculated K_d_’s followed a similar ranking to the enzymatic and TSA’s, with Idronoxil and NCGC00879727 the most potent, but whereas TSA failed with NCGC00418121 and NCGC00418159, we observed a K_d_ of >50 μM and 14.1 μM, using Spectral shift and MST, respectively.

### 2.7. Mechanistic insight of inhibition compared to ATP

We compared candidate compound activities in the ADP-Glo assay at 10 μM and 100 μM ATP conditions, compound potency shifts would indicate an ATP-competitive nature. As shown in Figs S7A–D and Table S11, an IC_50_ ratio range of 1.8 to 5.0 was observed among the compounds tested, with Idronoxil and NCGC00879727 appearing to be ATP-competitive. The weaker biochemical hits, NCG00418121 and NCGC00418159, were partial curves due to the competition leading to an estimated IC_50_ value.

### 2.8. Cellular evaluation of candidate ITPK1 inhibitors

**Fig. 5.**
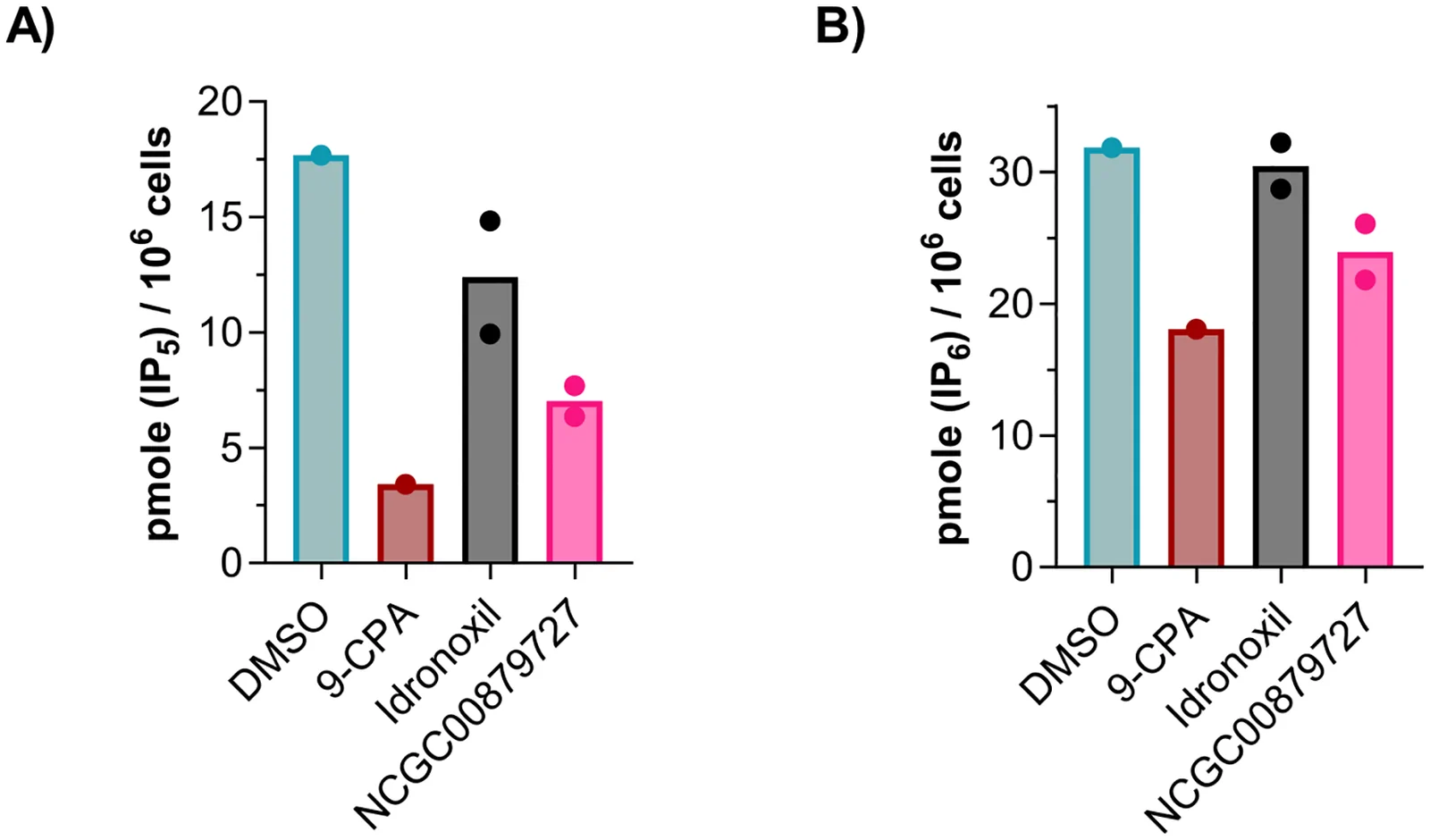
Effects of Idronoxil and NCGC00879727 on intracellular IP_5_ and IP_6_ levels. SW620 cells were cultured and treated with compounds for 6 days with refreshing compounds on day 4. Ten million live cells were then collected, and inositol phosphates were extracted for analyzing the amount of IP_5_ (A) and IP_6_ (B) by capillary electrophoresis coupled to electrospray ionization mass spectrometry. *y*-axis represents the amount of the analyzed IP in picomole per million cells and data points represent each independent experiment (n = 1 – 2).

To investigate ITPK1 inhibition in a cellular context, we tested Idronoxil and NCGC00879727 for their ability to reduce IP_5_ and IP_6_ levels in SW620 colon cancer cells, as previously described, using 9-CPA as a benchmark [5]. First, 9-CPA, Idronoxil and NCGC00879727 were tested in dose-response for six days to determine the maximum treatment concentration that does not affect cell viability (Fig. S8). We chose 10, 1, and 10 μM for 9-CPA, Idronoxil and NCGC00879727, respectively. After 6-day treatment with the indicated doses of compounds, cell extracts were analyzed for IP_5_ and IP_6_ levels by capillary electrophoresis electrospray ionization mass spectrometry. As shown in Fig. 5A and B, control 9-CPA performed as expected, reducing both IP_5_ and IP_6_ levels compared to DMSO control. While Idronoxil had little to no effect, NCGC00879727 decreased IP_5_ and IP_6_ levels, albeit not to the extent of 9-CPA, suggesting favorable cell-permeability and an ability to disrupt ITPK1’s ability to process inositol phosphates.

### 2.9. Structural insights into binding of NCGC00879727 to ITPK1

**Fig. 6.**
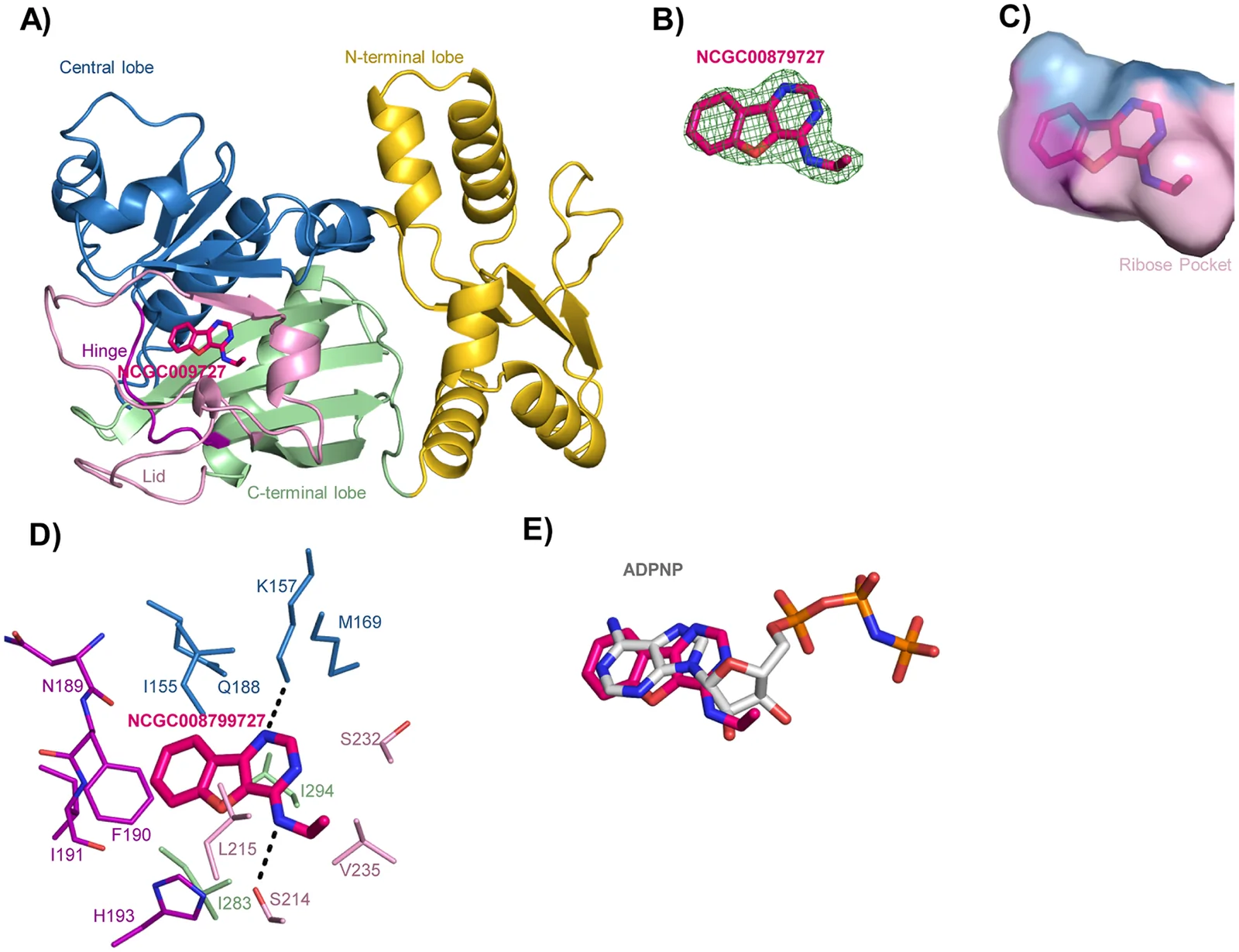
Crystal Structure of ITPK1 in Complex with Compound NCGC00879727. (A) Cartoon representation of the overall ITPK1–NCGC00879727 complex structure. The domain organization follows the nomenclature used for Zea mays ITPK1 [35]. The N-terminal lobe (including a helix from the C-terminus) is shown in gold, the central lobe in blue, and the C-terminal lobe in green. The hinge connecting the central and C-terminal lobes is purple. The lid region is colored pink. Compound NCGC00879727 is shown as sticks (carbon: hot pink, nitrogen: blue). (B). Fo–Fc electron density map (green mesh) contoured at 2.5σ shows clear density for compound NCGC00879727 (showing one of two chains). (C) Culled surface view of the inhibitor binding pocket, generated in PyMOL (detection radius: 7 Å; solvent radius cutoff: 5). (D) Close-up view of key residues (thin sticks) interacting with compound NCGC00879727 (thick sticks). Hydrogen bonds are shown as black dashed lines. (E) Superposition of ITPK1 structures bound to NCGC00879727 and ADPNP. The ADPNP molecule is shown with gray carbon, blue nitrogen, red oxygen, and orange phosphorus atoms.

Based on its activity profile, commercial availability, and structural tractability, we selected NCGC00879727 for further testing. To elucidate the inhibitory mechanism of compound NCGC00879727, we determined the crystal structure of the core catalytic domain of human ITPK1 in complex with NCGC00879727. There are two molecules per asymmetric unit. The difference electron density map revealed a well-defined unmodeled density within the ATP-binding pocket, which allowed unambiguous placement of NCGC00879727 (Fig. 6A and B). The structure of ITPK1 comprises three distinct lobes (Fig. 6A): an N-terminal lobe with an αβα fold and a helix from the C-terminus, a central lobe, and a C-terminal lobe connected by a hinge, forming the characteristic ATP-grasp fold. A lid region (residues 214–256) from the C-terminal lobe covers the nucleotide-binding site and overlaps with the binding site of the nucleoside moiety of ATP, which is now occupied by NCGC00879727 (Fig. 6A–C). NCGC00879727 forms two specific hydrogen bonds with residues Lys157 and Ser214. In addition, it engages in van der Waals interactions with multiple surrounding residues, including Ile155, Met169, Gln188, Asn189, Phe190, Ile191, His193, Leu215, Ser232, Val235, Ile283, and Ile294. The aminoethane moiety of NCGC00879727 extends into the ribose-binding pocket, a region we previously characterized (Fig. 6C). These observations indicate that further extension or optimization of the aminoethane moiety could improve inhibitor potency and/or selectivity. Together, these structural features illustrate the crystallographic binding mode of NCGC00879727, defining the interactions that dictate its potency and establishing a framework for future optimization (Fig. 6D–E, and Table S12).

## 3. Discussion

While ITPK1 is a pivotal player in inositol phosphate regulation, a high-quality chemical probe to study its role in disease would significantly enable research in this area. Although 9-CPA shows potent inhibition, its overall profile does not fully meet the criteria typically expected of a well-characterized chemical probe [4,36]. To identify new starting points suitable for medicinal chemistry optimization, we used a hybrid experimental and in silico strategy that integrates screening data with model-guided prioritization, an approach we have applied successfully in prior probe development campaigns [22,24,25,37]. In practice, this framework offers several advantages: it improves enrichment for true actives, helps preserve chemical diversity while reducing the number of compounds that must be tested experimentally, and supports earlier triage of liabilities such as weak efficacy or limited selectivity. Together, these features can shorten the path from initial screening to tractable lead series for probe optimization.

Historically, comparable efforts involved screening 400,000 compounds in qHTS format, generating millions of screening results. Here, we shifted to a model-informed workflow that screened approximately 20,000 compounds (20-fold less) experimentally and extended chemical space coverage through QSAR and machine-learning prioritization, while maintaining the same objective of identifying tractable starting points for probe development. This strategy was supported by a tiered experimental platform spanning high-, medium- and low-throughput assays for hit identification and characterization. To our knowledge, several elements of this suite have not been reported previously for ITPK1, including a scalable ADP-Glo (activity) and SDR (binding) assays. SDR offers practical advantages over comparable methods like intrinsic tryptophan fluorescence (ITF) [33], specifically by enabling binding assessment in cell lysates with reduced compound interference in 1536-well formats. We further show that SDR is applicable to kinases, providing an orthogonal binding readout to support biochemical inhibition data before progressing to cellular evaluation. Looking ahead, development of a higher-throughput cell-based assay that reports on ITPK1 activity will be important for advancing probe optimization and confirming cellular target engagement.

Our in silico workflow combined ligand-based QSAR modeling, ligand-based pharmacophore screening, and structure-based docking, each contributing a different filter on the same problem. QSAR captured patterns in the assay-confirmed data and enriched for compounds consistent with the SAR emerging from screening, while pharmacophore screening added a feature-based filter that was less dependent on scaffold identity and helped retain structurally distinct candidates that still shared the core features observed across active compounds. In parallel, docking provided a structure-based ranking that complemented the ligand-based approaches and offered a way to prioritize candidates that were not strongly represented in the training set. Using more than one method reduced reliance on any single model assumption and helped balance enrichment with chemical diversity during triage. Together, these approaches were used to interrogate a library of ~120,000 compounds. In a single iteration, the consensus ligand-based workflow increased the confirmed hit rate by ~58-fold relative to empirical screening and expanded the set of chemical series available for follow-up. That said, the experimentally tested set from the virtual screen was still modest, and potency gains were limited, with only a small fraction meeting the predefined potency and efficacy thresholds. Structure-based docking provided fewer active compounds in this campaign, reflecting the challenges of large-scale docking and scoring in a single target conformation. In future efforts, we expect that repeated cycles of model refinement and iterative cycles of virtual screening to explore ultra-large in silico libraries will be crucial for expanding chemical exploration and increasing the chances of finding higher-potency starting points.

Overall, our screening paradigm led to two compounds with ITPK1 inhibitory activity: (1) Idronoxil (CID 219,100) [38], which has been identified as a double-digit nanomolar inhibitor of ENOX2 (Ecto-NADH oxidase of tumor cell) along with acting as a modulator of DNA topoisomerase II. In tumor cells, ENOX2 is essential for maintaining the transmembrane electron balance and supporting cell growth. Idronoxil has been studied for its role in targeting pathways involved in cancer cell growth and survival [39]. The molecule is currently undergoing clinical trials to assess its safety and efficacy in cancer treatment [39]. Interestingly, by establishing Idronoxil as an ITPK1 inhibitor, our study uncovers a novel polypharmacological mechanism that may contribute to its broader clinical efficacy. (2) NCGC00879727 (CID 2877,580) has no publications and very little publicly available information on bioactivity, with only eight (inactive) results in PubChem [40]. Our crystal structure of ITPK1 bound to compound NCGC00879727 offers mechanistic insights into how the inhibitor competes with ATP at the nucleotide-binding site. NCGC00879727 establishes polar interactions with two key residues and is stabilized through extensive hydrophobic contacts within the ATP-grasp fold. The specific accommodation of the aminoethane group within the ribose pocket, shaped by the lid region, underscores the importance of steric complementarity for binding affinity. These findings provide a structural rationale for the selective inhibition of ITPK1 and lay the foundation for designing next-generation inhibitors with improved efficacy.

Idronoxil offers researchers an immediate tool compound to test ITPK1’s biological role while NCGC00879727 offers a (non-nucleoside) foundational scaffold to becoming a high-quality chemical probe [6,18,29]. Altogether, this study opens a new path for discovering ITPK1 inhibitors, which could be developed as pharmacological treatments for various human diseases, and has broad applicability to other inositol kinases.

## 4. Materials and methods

Protein Expression and Purification. The core catalytic domain of Human ITPK1 (ITPK1) (residues 1 to 335) [35], human inositol polyphosphate multikinase (IPMK) and the kinase domain of human diphosphoinositol pentakisphosphate kinase 2 (PPIP5K2, residues 1 to 366) [41] were cloned, expressed and purified as previously described [26]. Briefly, the constructs were cloned into a pDest566 vector containing a TEV protease cleavable maltose-binding protein tag (MBP). ITPK1 and IPMK were expressed by pGro/BL21 *Escherichia coli* cell. A 5 mL-culture in LB medium supplemented with 100 mg/mL ampicillin and 30 mg/mL chloramphenicol was grown for 6 h at 37 °C. The culture was then transferred into 1 L of 2xYT medium with 100 mg/mL ampicillin and 30 mg/mL chloramphenicol and 0.07% L-arabinose and incubated at 37 °C until an OD600 of 0.4–0.6 was reached. Then, the temperature lowered to 15 °C. After 1 h, 0.1 mM IPTG was added, and the cell culture was incubated at 15 °C for an additional 20 h. PPIP5K2 was expressed by ArcticExpress (DE3) *E. coli* cell. A 5 mL-culture in LB medium supplemented with 100 mg/mL ampicillin was grown for 6 h at 37 °C. The culture was then transferred into 1 L of 2xYT medium with 100 mg/mL ampicillin and incubated at 37 °C until an OD600 of 0.4–0.6 was reached. The temperature was lowered to 10 °C. After 1 h, 0.1 mM IPTG was added, and the cell culture was incubated at 10 °C for 3 days. Cells were harvested by centrifugation at 5000 x g for 10 min.

Cells were lysed by a constant cell disruption system (Constant Systems LTD) under 20,000 psi twice in a 20 mM Tris–HCl pH 7.2, 300 mM NaCl, and 20 mM imidazole buffer. The lysed cells were centrifuged at 13,000 x g at 4 °C for 45 min. The supernatant was mixed with 5 mL Ni-NTA agarose for 15 min at 4 °C and washed with 300 mL 20 mM Tris–HCl pH 7.2, 300 mM NaCl, and 20 mM imidazole buffer. The bound protein was eluted using a 20 mM Tris–HCl pH 7.2, 50 mM NaCl, and 250 mM imidazole buffer. The fusion product was further loaded on a HiTrap Heparin column (Cytivia) for PPIP5K2 and IPMK, and a HiTrap Q HP column (Cytivia) for ITPK1. The bound protein was eluted by using a 50 mM to 2000 mM NaCl gradient in a 20 mM Tris–HCl pH 7.2 buffer. The eluted protein was concentrated and further purified on a Superdex 200 (S200) Increase 10/300 GL column (Cytivia) in a 20 mM HEPES pH 7.2 and 150 mM NaCl buffer. The nonaggregate protein was pooled and cleaved by incubation in 5 mM DTT and 0.01 mg/mL TEV overnight at 4 °C. MBP tag was separated on a HiTrap Heparin column for PPIP5K2 and IPMK, and a HiTrap Q HP column for ITPK1. The cleaved proteins were purified again on the S200 column. Proteins (Fig. S9) were stored at −80 °C. For crystallization, a construct of human ITPK1 (ITPK1) with residues 1 to 328 was cloned and purified.

Synthesis of Inositol Phosphates. All materials were purchased from commercial sources and used without further purification unless otherwise noted. Detailed description of synthesis is shown in Supplementary Information Material and Methods.

1536-well Assay Optimization. Based on the results from the 96-well format, we proceeded to miniaturize the assay to the 1536-well format. We chose the ADP-Glo Kit from Promega due to its high sensitivity and success of running at our screening facility. To reduce costs, we initially attempted a cost-effective fluorescence-based coupled assay but after multiple attempts, a minimal signal was observed and this method was abandoned [42]. Similar to the above kit, ADP-Glo is a bioluminescent assay that depletes ATP leaving the remaining ADP to be converted into a luminescence signal [11]. This would allow us to monitor the catalysis of (3,4,5,6)P_4_ to IP_5_, and IP_7_ to IP_8_ for ITPK1 and PPIP5K2, respectively. First, we miniaturized the above 25 μL assay in 96-well format to 4 μL in 1536-well format, modifying the assay buffer to include 0.01% Tween20, starting with 10 μM ATP (K_m_ = 10 – 22 μM) and substrate concentrations 1 μM 3,4,5,6-IP_4_ (K_m_ = 0.1 μM) [43] and 1 μM 5-IP_7_ (K_m_ = 0.06 μM) [44] for ITPK1, and PPIP5K2, respectively. We tested ITPK1 and PPIP5K2 at concentrations of 25, 50, 100, 200 nM, and at time-points of 5, 10, 15, 30, 60, 120 and 240 min to examine the optimized condition for 1536-well format. Based on statistical analysis from iterative experiments with varied ITPK1 or PPIP5K2 concentrations, 100 nM of enzyme was chosen for our initial HTS conditions, exhibiting a S:B > 10 and Z’-factor>0.8 for a robust HTS assay (Fig. S1). Assay performance (Z’-factor) was calculated from the change in luminescence intensity over the respective reaction period was normalized against no-inhibitor or no-enzyme for neutral (DMSO) or positive controls, respectively. An incubation time of 60 min was chosen, representing ~88% and 73% conversion for ITPK1 and PPIP5K2, respectively, balancing the ability to identify inhibitors with assay performance [14,45–47]. Over the time-course, RLU, S:B, and Z’ values increased proportionally over the concentrations and times tested, for both enzymes, respectively (Figs S1C – D). At ≥60 min, the S:B value was relatively stable at ≥100 nM ITPK1, with Z’ values >0.8. In contrast, for PPIP5K2 an increase in both RLU and S:B was observed over the time-course, but Z’-factor plateaued at ≥50 nM and ≥60 min, with Z’ values >0.8.

Next, to minimize ADP-Glo usage, we tested ITPK1 assay with 0.5, 1, and 2 μL of ADP-Glo reagent and 1, 2, and 4 μL of Detection reagent (Table S13). Assay statistics were consistent with S:B ~11 and Z’ ~0.7; we chose 0.5 or 1 μL of ADP-Glo reagent, and 1 or 2 μL of Detection reagent moving forward. Final reaction conditions were: dispensed (BioRaptr 2.0, Let’s Go Robotics) 3 μL of enzyme mixture (final concentrations of 50 or 100 nM ITPK1) and assay buffer (25 mM Tris–HCl pH 7.5, 5 mM MgSO_4_, 0.1 mM EDTA pH 8.0, 0.1 mM NaN_3_, with 0.01% Tween20) into columns 1 – 2, 5 – 48, and columns 3 and 4, respectively, of a 1536-well solid-bottom medium binding white plate (Greiner Bio-One, Monroe, NC). Next, 20 or 40 nL of DMSO or compound (final concentration range 389 nM to 99 μM) or intraplate control NDGA (NCGC00015741 final concentration range 12.1 nM to 99 μM) was transferred via acoustic droplet ejection (Echo Liquid Handler, Labcyte, Indianapolis, IN) and incubated (RT) for ~15 min. Next, 1 μL of substrate mixture containing 3,4,5,6-IP_4_ and ATP (final concentrations of 1 μM and 10 μM, respectively) was dispensed, and the reaction proceeded for 60 min (RT, protected from light) followed by a 0.5 or 1 μL addition of ADP-Glo Reagent^®^. Samples were incubated (RT, protected from light) for 30 min to terminate the enzyme reaction and remove the remaining ATP, followed by a 1 or 2 μL addition of Kinase Detection Reagent ^®^ to allow for ADP to be converted to ATP, which was then detected with a luciferase reaction. Samples were centrifuged (Eppendorf 5804R) for 15 s at 1000 RPM (164 x g) followed by incubation at room temperature for 15 to 30 min. Plates were then read using Luminescence optics on a ViewLux High-throughput CCD imager (PerkinElmer, Waltham, MA).

Compounds, NCATS Screening Collection, High-throughput Screening Statistics, and Assay Performance. Screened libraries [19] included MIPE 5.0 (2418 compounds) [15], Genesis Minime [21], Genesis [20], and Artificial Intelligence Driven (AID) Library (6995 compounds) [22]. For follow up confirmatory assays, all compounds were sourced as fresh solutions from NCATS Compound Management.

Compounds from MIPE 5.0 and Genesis Minime were screened in qHTS format (5-concentrations points, 1:5 dilution; final assay concentration of 2 nM to 100 μM; ≤1% DMSO final), while AID was screened at a single concentration of 50 μM final. The assay performance across the 50 1536-well plates We observed an excellent mean Z’-factor of 0.84 ± 0.13 (Fig. 2A). For the Genesis Minime and AID screens, we calculated the Minimum Significant Ratio (MSR), a statistical parameter that characterizes the reproducibility of potency estimates from in vitro concentration-response (CRC) assays. The intraplate control NDGA performed well, with mean IC_50_’s [μM] of 2.80 ± 1.08 μM (MSR = 2.3; Fig. S10A), and 1.15 ± 0.20 μM (MSR = 1.6; Fig. S10B), respectively, indicating strong assay reproducibility [48]. Screening in qHTS format yields CRCs from the primary screen, enabling us to classify actives as samples yielding a curve class (CC) of −1.1, −1.2, −2.1, −2.2 [7,49–52]. Selectivity was defined as being active towards the isozyme of interest and inactive against the other isozymes and/or exhibiting a > 5-fold IC_50_ ratio if activity was observed.

Radiometric inositol phosphate kinase assay. The ITPK1 kinase reaction was initiated by mixing 0.26 nM ITPK1, 10 μM ATP, 1 μM [33P] Ins(3,4,5,6)IP_4_ and incubating at 37 °C for 60 min in the ITPK1 reaction buffer (20 mM HEPES pH 7.2, 100 mM KCl, 0.01% TritonX-100, 3 mM MgCl_2_, 50 μM EDTA and 1 mM DTT). The kinase activities of PPIP5K2 and IPMK were also evaluated in the presence of ITPK1 inhibitors. For PPIP5K2 kinase assay, 5 nM PPIP5K2 was incubated with 10 μM ATP, 1 μM [33P]5-IP_7_ (40,000 dp.m.), and 100 μM ITPK1 inhibitors at 37 °C for 60 min. For the IPMK kinase assay, 200 nM IPMK was incubated with 10 μM ATP, 1 μM [33P](1,3,4,5)P_3_ (80,000 dp.m.), and 10 μM ITPK1 inhibitors at 37 °C for 60 min. All assays containing radiolabels were quenched with 150 mM NH_4_H_2_PO_4_, pH 3.9 and 40 mM EDTA, and analyzed by ion-exchange HPLC, using a 4.6 × 125 mm, 5 μm Parti-Sphere SAX column. The elution gradient (1 mL/min) was generated by mixing Buffer A (1 mM EDTA) with Buffer B (1 mM Na2EDTA, 2.5 M NH_4_H_2_PO_4_, pH 3.9); the elute was mixed with 1 mL/min Monoflow4 scintillation liquid (National Diagnostics) and radioactivity was monitored in-line with a Beta-RAM 6 radio flow detector (LabLogic).

Protein Thermal Shift Assay (TSA). Thermal stability of ITPK1 was assessed in the presence of compounds as described previously, with modifications [29,30,53]. Briefly, His-tagged ITPK1 was diluted to a final concentration of 2 μM in assay buffer (50 mM HEPES pH 7.5, 5 mM MgSO_4_, 0.1 mM EDTA pH 8.0, 150 mM NaCl, 0.001% Triton X-100) containing 8X SYPRO orange dye (Invitrogen). The final assay volume was 10 μL in a 384-well PCR plate (Roche LightCycler^®^ 480 Multiwell Plate, white). Compounds were transferred (100, 200, or 400 nL) via acoustic dispenser (Echo 555, Labcyte) for a final concentration range of 1.56 to 200 μM. This was followed by centrifugation (Eppendorf 5804R) for 15 s at 1000 RPM (164 x g), sealed (LightCycler^®^ 480 Sealing Foil), then read on a Roche LightCycler 480 II using standard DSF conditions (20–85 °C, 0.05 °C/s, 10 acquisitions per °C) for fluorescence using SYPRO orange optics (E_x_ = 465 / E_m_ = 580) [30]. Target unfolding parameters (T_m_) were derived from the first derivative curves using Roche Thermal Shift Analysis (v1.5.1) and GraphPad Prism (v10.2.3) software. To identify false positives, a counterscreen was performed under identical conditions with the protein replaced by buffer. Compounds showing significant fluorescence transitions in the absence of protein were classified as assay interference artifacts and excluded (Fig. S5). Alternative methods to mitigate interference, including Nile Red (E_x_ = 559 / E_m_ = 636) [54–56], nanoDSF [30], or Cy5-Tris NTA labeling (E_x_ = 648 / E_m_ = 671) [34] were evaluated but yielded unsatisfactory results (data not shown).

Microscale Thermophoresis (MST) or Spectral Shift protein and small molecule binding. The binding affinity of the compounds to (His)_6_-ITPK1protein (full-length; N-terminal His-tag purchased from MyBioSource) was evaluated using microscale thermophoresis (MST) or Spectral Shift on a Monolith X (Nanotemper Technologies). Recombinant protein was labeled with a fluorophore using Monolith His-tag labeling RED-tris-NTA 2nd Generation kit (Nanotemper Technologies or HIS Lite^™^ Cy5 Tris NTA-Ni Complex from AAT Bioquest) following the manufacturer’s protocol in assay buffer (50 mM HEPES pH 7.5, 5 mM MgSO_4_, 150 mM NaCl, and 0.1% Pluronic F-127). Compounds were transferred (100 nL) via acoustic droplet ejection (Echo 650) from the previously prepared confirmatory plates (10 mM, 1:2 or 1:3 dilution, 11-points; final concentration range of 1.7 nM to 100 μM) and incubated with the same volume of 100 nM (final concentration; 50 nM RED-tris-NTA) labeled recombinant protein for >5 min at RT (10 μL final assay volume). Measurements (single experiment, measured n = 4 or n = 5) were carried out in assay buffer (50 mM HEPES pH 7.5, 5 mM MgSO_4_, 150 mM NaCl, and 0.1% Pluronic F-127) and premium capillaries using Monolith X instrument (Nanotemper Technologies, Munich, Germany) with 100% LED excitation power, IR laser on High power, MST before time of 3 *sec*, on-time of 30 *sec* and off-time of 1 *sec* (Fig. S6G - K). The dissociation constant (K_d_) values were calculated by fitting the thermophoresis signal at 15 *sec* of the thermograph using the MO Control software (v2.6.6; Nanotemper Technologies, Munich, Germany) and confirmed using Graphpad Prism (v10.4.2). Fnorm[%] values were converted to ΔFnorm[%] in Graphpad Prism using Baseline correction of the lowest concentration values (Value – Baseline) as shown in Figs S6A – G. Of note, in the representative plots aggregation was observed in some samples (Figs S6G and K, as examples).

Structural Dynamic Response (SDR) Assay [28]. The ITPK1 open reading frame was generated as either an N-terminal or C-terminal in-frame fusion to HiBiT tag containing a GS linker on either end (GSVSGWRLFKKISGS) by gene synthesis (GenScript) and cloned into the NheI/EcoRI sites of pcDNA3.1 (+). For lysate preparation, HEK293 cells (10 × 10^6^ in 10 mL) were transfected with ITPK1-tagged constructs (20 μg DNA plus 45 μg Lipofectamine 2000 in OptiMEM) using a reverse transfection procedure and seeded into a T75 flask. Cells were harvested after 24 h and resuspended at 1 × 10^6^ cells/mL in SDR assay buffer (DPBS with CaCl_2_ and MgCl_2_ containing 1 g/L glucose and 1 × Halt Protease inhibitor cocktail, ThermoFisher). Cells were lysed with the addition of NP40 at a final concentration of 0.4%. Following a 30 min room temperature incubation, lysate was clarified at 3200 g for 10 mins (4 °C) to remove insoluble material, then the supernatant was transferred to a 50 mL conical tube. Lysates were first tested for expression level of each construct (Fig. S4A). Subsequently, 9-CPA or DMSO were predispensed (20 nL) onto 1536-well Aurora white cyclic olefin, high solid base, medium bind plates. Four μL/well buffer control or HiBiT-tagged ITPK1 were dispensed with a BioRaptr (Let’s Go Robotics, Inc.) followed by centrifugation (1000 rpm or 164 g) for 10 s and a 30 minute incubation at room temperature protected from light. The Nano-Glo substrate solution was prepared according to manufacturer’s protocol where LgBiT was added at 1:100 dilution and NLuc substrate (furimazine) was prepared at 1:50 dilution in Nano-Glo HiBiT lytic buffer (Promega). The substrate solution was added to all wells at 2 μL/well, incubated at room temperature for 10 min, then read on a ViewLux CCD plate reader for luminescence. Because of the better dynamic range observed for 9-CPA (Fig. S4B), we proceeded to test the candidate inhibitors in lysates expressing N-terminally tagged ITPK1. The lysate was then diluted in SDR buffer to obtain a basal signal between 100 – 2000 RLUs (1:32 dilution; Fig. S4C) and candidate inhibitors were tested as above.

The counterscreen was performed by first predispensing (20 nL) compound onto 1536-well Aurora white cyclic olefin, high solid base, medium bind plates, followed by dispensing 5 μL/well of 86b peptide (final concentration 1 pM) or SDR assay buffer. Following a 30 min room temperature incubation, 3 μL of Nano-Glo substrate solution was added to all wells, incubated (RT) for 10 min, then read on a ViewLux CCD plate reader for luminescence.

Crystallization, Data Collection, Structure Determination, and Analysis. Crystals of apo ITPK1 (residues 1–328) were obtained by the hanging-drop vapor diffusion method at 4 °C over the course of one week. Each hanging drop consisted of 2 μL of protein/ligand solution mixed with 2 μL of reservoir buffer, which contained 21% (w/v) PEG 8000, 0.17 mM (NH_4_)_2_ SO_4_, 85 mM Tris–HCl (pH 8.0), and 10% glycerol. To generate inhibitor-bound complexes, crystals were soaked in a buffer containing 25% (w/v) PEG 8000, 0.17 mM (NH_4_)_2_SO_4_, 85 mM Tris–HCl (pH 8.0), and 20% glycerol. Diffraction data were collected at the NSLS-II (Brookhaven National Laboratory) using beamline AMX. The structure was solved by molecular replacement using the Phaser-MR program within the PHENIX suite [57], with PDB entry 2ODT serving as the search model. Model building was performed manually using COOT [58], and refinement was carried out with PHENIX. Molecular graphics were prepared using PyMOL (Schrödinger, LLC). Atomic coordinates and structure factors have been deposited in the Protein Data Bank under accession code 13LZ (see Supplementary Table S12).

Machine Learning Modeling. We developed and applied a deep learning consensus architecture (DLCA [59]) classification model to predict ITPK1 inhibitory activity. The model was built using 486 compounds from our screening campaign, of which 81 were actives and 405 were inactives. To avoid noise from borderline or inconsistent measurements, only high-confidence annotations were retained. Active compounds were defined as compounds that were active in both the primary and follow-up ITPK1 assays and inactive in the ADP-Glo counterscreen. Compounds with weak or ambiguous activity profiles, including those near the efficacy threshold or with borderline curve classifications, were excluded. Because the number of inactive compounds substantially exceeded the number of actives, a structurally diverse subset of inactive compounds was selected for model development. This approach reduced class imbalance, avoided over-representation of closely related inactive chemotypes, and maintained broad coverage of the inactive chemical space. PPIP5K2 selectivity data were not used as model labels but were used after experimental testing to prioritize ITPK1-selective compounds. The dataset was divided into a training set containing 80% of the compounds (65 actives and 323 inactives) and an external validation set containing the remaining 20% (16 actives and 82 inactives).

The DLCA integrated predictions from multiple deep neural networks built on different molecular representations, including Morgan, Avalon, and AtomPair fingerprints, along with RDKit-based physicochemical descriptors. We also included a convolutional neural network trained directly on SMILES strings to capture information embedded in molecular connectivity. Averaging the predictions from these models produced a consensus score that balanced the strengths of each representation and minimized bias from any single approach.

To avoid training-set leakage during prospective virtual screening, compounds used for DLCA model development were excluded from the Genesis collection prior to model application. The trained model was then applied to the remaining compounds in the collection. Candidate compounds for experimental testing were selected from the overlap between DLCA-predicted actives and compounds with high pharmacophore fit scores.

### 4.1. Pharmacophore (PH4) virtual screening

Ligand-based pharmacophore (LBP) models were generated in LigandScout 4.4 Advanced (Inte:Ligand GmbH) [60] using the most potent, confirmed ITPK1 inhibitors from our screening campaign. We selected the top 7 compounds with IC_50_ values below 20 μM (Table S8). These molecules were clustered using pharmacophore-based similarity thresholds of 0.4, 0.6, 0.7, and 0.8 to group related chemotypes. For each cluster, we generated both merged-features pharmacophore (MFP) and shared-features pharmacophore (SFP) models. The MFP combines all pharmacophore features from different compounds in a cluster into a single model, incorporating overlapping features from different ligands. The SFP captures only the pharmacophore features consistently present across all ligands in the cluster [61]. The MFP is well-suited for capturing broader SAR variation, while the SFP focuses on conserved features critical for biological activity. Both model types were evaluated for their ability to correctly identify actives over inactives. Each model was evaluated against the complete set of confirmed actives and inactives, and the proportion of each correctly identified was calculated. Models that recovered at least 20% more actives than inactives were kept for virtual screening.

Given the abstract nature of pharmacophore models, they provide an efficient approach for virtually screening extensive compound libraries [62]. The final validated pharmacophore models captured key spatial arrangements of hydrogen-bond donors, hydrogen-bond acceptors, hydrophobic and aromatic regions, as well as exclusion volumes to represent steric constraints. These models were then applied to the broader Genesis library to identify compounds that matched the pharmacophore hypotheses. Compounds that matched the validated pharmacophore features were shortlisted, with the highest priority given to those that also scored well in the machine learning predictions.

Cellular Evaluation of Candidate ITPK1 Inhibitors. Human cancer cell lines SW620 were grown and maintained in McCoy’s 5A medium (Cytiva) supplemented with 10% fetal bovine serum (Gibco) and 100 units/mL of penicillin and 100 μg/mL of streptomycin (Cytiva #40,003.01). Cells were cultured at 37 °C in a humidified 5% CO_2_ incubator and were authenticated by using short tandem repeat DNA profiling (LabCorp) along with being frequently tested for mycoplasma using the MycoAlert PLUS Detection Kit (Lonza). To assess cell viability and proliferation, cells were seeded in 384-well plates and incubated overnight. The following day, compounds were added at the desired final concentrations. After three days of treatment, wells were replenished with fresh compound to maintain the final concentrations, and cells were incubated for an additional two days. Cell viability was determined by using CellTiter-Glo assay (Promega) and normalized against non-treated conditions. Cell proliferation was analyzed by monitoring changes in cell confluence using IncuCyte Live-Cell Analysis System (Sartorius). For Inositol phosphate extraction, 250,000 cells were plated in 10-cm dishes (CytoOne) and cultured overnight. Next day, culture media was replaced with fresh media containing the 10 μM of 9-CPA and NCGC00879727, and 1 μM of Idronoxil with DMSO as a negative control. Cells were treated for six days with the compound-containing media being refreshed on the fourth day of the treatment. After the six-day treatment with compounds, cells were trypsinized and resuspended for live cell counting by using Trypan Blue staining (Gibco #15,250–061). Live cells (~1 × 10^6^) were collected, washed once with ice-cold DPBS (Corning), pelleted, and stored in a −80 °C freezer. Inositol phosphates were extracted by following previously established protocols [63,64]. Briefly, cell pellets were lysed in 1 M perchloric acid (Millipore Sigma) on ice for 30 min with brief vortexing at five minute intervals. Lysates were then clarified by centrifugation (12,000 rpm) at 4 °C for 5 min, then the soluble fraction was transferred to a clean tube and incubated with 5 mg PCA-washed TiO_2_ beads (GL Sciences) at 4 °C with rotation for 30 min. Beads were washed three times with ice-cold 1 M perchloric acid at 4 °C with rotation for 5 min each and pelleted by centrifugation (12,000 rpm) at 4 °C for 5 min. To elute inositol phosphates from the beads, pelleted beads were incubated with 800 μL of 10% NH_4_OH (Millipore Sigma) with rotation for 30 min, and once again with 400 μL of 10% NH_4_OH for another 30 min. Eluents were combined and dried using a speed vacuum concentrator. Dried samples were stored in a −80 °C freezer until being analyzed by capillary electrophoresis electrospray ionization mass spectrometry [5,65].

### 4.2. Structure-based molecular docking

We used the crystal structure of human ITPK1 (PDB ID: 2Q7D) [4] prepared in Maestro for docking studies [66]. Protein preparation was done using the Protein Preparation Wizard [67–71], which involved removing crystallographic waters and non-essential heteroatoms, adding hydrogens, assigning protonation states for physiological pH, and carrying out restrained minimization to optimize side-chain geometry in the binding site. Ligands from the Genesis library were prepared in Maestro using LigPrep [72]. This included generating 3D conformations, assigning appropriate protonation states, and minimizing each ligand structure. We first docked the full Genesis collection using standard-precision (SP) mode [73,74]. The top 10,000 scoring compounds from the SP run were then re-docked in extra-precision (XP) mode [73, 74]. From the XP results, we took all compounds with docking scores less than −11 kcal/mol, giving us 47 high-scoring candidates. To maintain chemical diversity while still capturing reasonable binders, we also included compounds with XP scores less than −8 kcal/mol, which produced ~1600 additional candidates. We then applied RDKit’s diversity picker to this set and selected 81 diverse representatives. Combining these with the top 47 gave us a final set of 128 compounds for experimental testing. qHTS data analysis. Data from each assay were normalized plate-wise to corresponding intra-plate controls (neutral DMSO and positive control), which were also used to calculate the Z’ factor for assay quality control. Percent activity, Concentration-response curves (CRCs) fitting and classification, and IC_50_ determination were performed using in-house software as previously described [7,48–51,75]. CRCs were fitted and classified as previously described, categorized into four classes: complete response curves (class 1), partial curves (class 2), single point actives (class 3), and inactives (class 4). Otherwise, concentration–response curves were fitted and IC_50_ values were calculated using Prism software (version 10, GraphPad Software), sigmoidal dose-response (variable slope). Minimum Significant Ratio (MSR), a statistical parameter that characterizes the reproducibility of potency estimates from in vitro CRC assays, was used to assess the performance of our intraplate controls. The chemical structures were standardized using the LyChI (Layered Chemical Identifier; normalized charge) program (version 20,141,028, https://github.com/ncats/lychi). Hit selection criteria were aggregated for duplicate structures using LyChi-3 provided by the NCATS Resolver. This was all done within the Palantir Technologies Foundry Platform (Washington, DC), which is configured to ingest all HTS results generated at NCATS and harmonized this data with other sources such as ChEMBL and OrthoMCL. All qHTS screening results are publicly available at PubChem (https://pubchem.ncbi.nlm.nih.gov/source/NCGC) AIDs: 2202,724, 2202,725, 2202,726, and 2202,727.

## Supplementary Material

mmc1

mmc2

mmc3

Supplementary material associated with this article can be found, in the online version, at doi:10.1016/j.slasd.2026.100323.

## References

[1] Wang H Structural insights into the development of inhibitors for inositol phosphate kinases. FEBS Lett 2026. 10.1002/1873-3468.70280. Jan 20. PMC12823050 41559740

[2] Krober T, Bartsch SM, Fiedler D. Pharmacological tools to investigate inositol polyphosphate kinases - enzymes of increasing therapeutic relevance. Adv Biol Regul 2022;83:100836. 10.1016/j.jbior.2021.100836. Jan. 34802993

[3] Richterich CA, Logtenberg MEW, Jansen MJH, Toebes M, Bresser K, Borst A, Jurgens AP, Leusen JHW, Schumacher TN. ITPK1 Sensitizes tumor cells to IgA-dependent neutrophil killing In vivo. J Immunol 2024;213(8):1244–54. 10.4049/jimmunol.2300372. Oct 15. 39213127 PMC7616474

[4] Chamberlain PP, Qian X, Stiles AR, Cho J, Jones DH, Lesley SA, Grabau EA, Shears SB, Spraggon G. Integration of inositol phosphate signaling pathways via human ITPK1. J Biol Chem 2007;282(38):28117–25. 10.1074/jbc.M703121200. Sep 21. 17616525 PMC2244811

[5] Ng MY, Wang H, Zhang H, Prucker I, Perera L, Goncharova E, Wamiru A, Jessen HJ, Stanley RE, Shears SB, Luo J, O’Keefe BR, Wilson BAP. Biochemical and biophysical characterization of inositol-tetrakisphosphate 1-kinase inhibitors. J Biol Chem 2025;301(3):108274. 10.1016/j.jbc.2025.108274. Mar. 39922495 PMC11927698

[6] Sanfelice D, Antolin AA, Crisp A, Chen Y, Bellenie B, Brennan PE, Edwards A, Muller S, Al-Lazikani B, Workman P. The Chemical Probes Portal - 2024: update on this public resource to support best-practice selection and use of small molecules in biomedical research. Nucleic Acids Res 2025;53(D1):D1663–9. 10.1093/nar/gkae1062. Jan 6. 39558166 PMC11701680

[7] Inglese J, Auld DS, Jadhav A, Johnson RL, Simeonov A, Yasgar A, Zheng W, Austin CP. Quantitative high-throughput screening: a titration-based approach that efficiently identifies biological activities in large chemical libraries. Proc Natl Acad Sci U S A 2006;103(31):11473–8. 10.1073/pnas.0604348103. Aug 1. 16864780 PMC1518803

[8] Shockley KR, Gupta S, Harris SF, Lahiri SN, Peddada SD. Quality control of quantitative high throughput screening data. Front Genet 2019;10:387. 10.3389/fgene.2019.00387. 31143201 PMC6520559

[9] Lynch C, Sakamuru S, Ooka M, Huang R, Klumpp-Thomas C, Shinn P, Gerhold D, Rossoshek A, Michael S, Casey W, Santillo MF, Fitzpatrick S, Thomas RS, Simeonov A, Xia M. High-throughput screening to advance In vitro toxicology: accomplishments, challenges, and future directions. Annu Rev Pharmacol Toxicol 2024;64:191–209. 10.1146/annurev-pharmtox-112122-104310. Jan 23. 37506331 PMC10822017

[10] Wormald M, Liao G, Kimos M, Barrow J, Wei H. Development of a homogenous high-throughput assay for inositol hexakisphosphate kinase 1 activity. PLoS One 2017;12(11):e0188852. 10.1371/journal.pone.0188852. 29186181 PMC5706701

[11] Zegzouti H, Zdanovskaia M, Hsiao K, Goueli SA. ADP-glo: a bioluminescent and homogeneous ADP monitoring assay for kinases. Assay Drug Dev Technol 2009;7(6):560–72. 10.1089/adt.2009.0222. Dec. 20105026

[12] Puhl-Rubio AC, Stashko MA, Wang H, Hardy PB, Tyagi V, Li B, Wang X, Kireev D, Jessen HJ, Frye SV, Shears SB, Pearce KH. Use of protein kinase-focused compound libraries for the discovery of new inositol phosphate kinase inhibitors. SLAS Discov 2018;23(9):982–8. 10.1177/2472555218775323. Oct. 29842835 PMC6148399

[13] Hostachy S, Wang H, Zong G, Franke K, Riley AM, Schmieder P, Potter BVL, Shears SB, Fiedler D. Fluorination influences the bioisostery of myo-inositol pyrophosphate analogs. Chemistry 2023;29(67):e202302426. 10.1002/chem.202302426. Dec 1. 37773020 PMC7615343

[14] Brooks HB, Geeganage S, Kahl SD, Montrose C, Sittampalam S, Smith MC, Weidner JR, Basics of enzymatic assays for HTS. In: Markossian S, Grossman A, Brimacombe K, et al., editors. Assay guidance manual; 2004.

[15] Lin GL, Wilson KM, Ceribelli M, Stanton BZ, Woo PJ, Kreimer S, Qin EY, Zhang X, Lennon J, Nagaraja S, Morris PJ, Quezada M, Gillespie SM, Duveau DY, Michalowski AM, Shinn P, Guha R, Ferrer M, Klumpp-Thomas C, Michael S, McKnight C, Minhas P, Itkin Z, Raabe EH, Chen L, Ghanem R, Geraghty AC, Ni L, Andreasson KI, Vitanza NA, Warren KE, Thomas CJ, Monje M. Therapeutic strategies for diffuse midline glioma from high-throughput combination drug screening. Sci Transl Med 2019;11(519). 10.1126/scitranslmed.aaw0064. Nov 20. PMC7132630 31748226

[16] Zhang JH, Kang ZB, Ardayfio O, Ho PI, Smith T, Wallace I, Bowes S, Hill WA, Auld DS. Application of titration-based screening for the rapid pilot testing of high-throughput assays. J Biomol Screen 2014;19(5):651–60. 10.1177/1087057113512151. Jun. 24246376

[17] Huang R, Xia M, Cho MH, Sakamuru S, Shinn P, Houck KA, Dix DJ, Judson RS, Witt KL, Kavlock RJ, Tice RR, Austin CP. Chemical genomics profiling of environmental chemical modulation of human nuclear receptors. Environ Health Perspect 2011;119(8):1142–8. 10.1289/ehp.1002952. Aug. 21543282 PMC3237348

[18] Muller S, Ackloo S, Arrowsmith CH, Bauser M, Baryza JL, Blagg J, Bottcher J, Bountra C, Brown PJ, Bunnage ME, Carter AJ, Damerell D, Dotsch V, Drewry DH, Edwards AM, Edwards J, Elkins JM, Fischer C, Frye SV, Gollner A, Grimshaw CE, IJ A, Hanke T, Hartung IV, Hitchcock S, Howe T, Hughes TV, Laufer S, Li VM, Liras S, Marsden BD, Matsui H, Mathias J, O’Hagan RC, Owen DR, Pande V, Rauh D, Rosenberg SH, Roth BL, Schneider NS, Scholten C, Singh Saikatendu K, Simeonov A, Takizawa M, Tse C, Thompson PR, Treiber DK, Viana AY, Wells CI, Willson TM, Zuercher WJ, Knapp S, Mueller-Fahrnow A. Donated chemical probes for open science. Elife 2018;7:7. 10.7554/eLife.34311. Apr 20. PMC5910019 29676732

[19] https://ncats.nih.gov/research/research-activities/compound-management/chemical-libraries.

[20] Clausse V, Fang Y, Tao D, Tagad HD, Sun H, Wang Y, Karavadhi S, Lane K, Shi ZD, Vasalatiy O, LeClair CA, Eells R, Shen M, Patnaik S, Appella E, Coussens NP, Hall MD, Appella DH. Discovery of novel small-molecule scaffolds for the inhibition and activation of WIP1 phosphatase from a RapidFire mass spectrometry high-throughput screen. ACS Pharmacol Transl Sci 2022;5(10):993–1006. 10.1021/acsptsci.2c00147. Oct 14. 36268125 PMC9578142

[21] Hanson QM, Hoxie N, Shen M, Guo H, Cho IJ, Chakraborty I, Aragon BM, Rai G, Patnaik S, Janiszewski JS, Hall MD. Target class profiling of small-molecule methyltransferases. ACS Chem Biol 2023;18(4):969–81. 10.1021/acschembio.3c00124. Apr 21. 36976909 PMC10983791

[22] Yasgar A, Jain S, Davies M, Danchik C, Niehoff T, Ran J, Rai G, Yang SM, Simeonov A, Zakharov AV, Martinez NJ. Integrated approach of machine learning and high-throughput screening to identify chemical probe candidates targeting aldehyde dehydrogenases. ACS Pharmacol Transl Sci 2025;8(10):3568–84. 10.1021/acsptsci.5c00395. Oct 10. 41098570 PMC12519302

[23] Michael S, Auld D, Klumpp C, Jadhav A, Zheng W, Thorne N, Austin CP, Inglese J, Simeonov A. A robotic platform for quantitative high-throughput screening. Assay Drug Dev Technol 2008;6(5):637–57. 10.1089/adt.2008.150. Oct. 19035846 PMC2651822

[24] Hochuli JE, Jain S, Melo-Filho C, Sessions ZL, Bobrowski T, Choe J, Zheng J, Eastman R, Talley DC, Rai G, Simeonov A, Tropsha A, Muratov EN, Baljinnyam B, Zakharov AV. Allosteric binders of ACE2 are promising Anti-SARS-CoV-2 agents. ACS Pharmacol Transl Sci 2022;5(7):468–78. 10.1021/acsptsci.2c00049. Jul 8. 35821746 PMC9236207

[25] Jain S, Talley DC, Baljinnyam B, Choe J, Hanson Q, Zhu W, Xu M, Chen CZ, Zheng W, Hu X, Shen M, Rai G, Hall MD, Simeonov A, In Zakharov AV Hybrid. Silico approach reveals novel inhibitors of multiple SARS-CoV-2 variants. ACS Pharmacol Transl Sci 2021;4(5):1675–88. 10.1021/acsptsci.1c00176. Oct 8. 34608449 PMC8482323

[26] Wang H, Shears SB, Blind RD. Structural rationalization of IPMK inhibitor potency. J Med Chem 2025;68(22):24316–25. 10.1021/acs.jmedchem.5c02314. Nov 27. 41237254 PMC12670395

[27] Rothenaigner I, Hadian K. Brief guide: experimental strategies for high-quality hit selection from small-molecule screening campaigns. SLAS Discov 2021;26(7):851–4. 10.1177/24725552211008862. Aug. 33882754 PMC8293735

[28] Ciulla DA, Dranchak PK, Aitha M, van Neer RHP, Shah D, Tharakan R, Wilson KM, Wang Y, Braisted JC, Inglese J. A general assay platform to study protein pharmacology using ligand-dependent structural dynamics. Nat Commun 2025;16(1):4342. 10.1038/s41467-025-59658-6. May 10. 40346061 PMC12064818

[29] Wu T, Hornsby M, Zhu L, Yu JC, Shokat KM, Gestwicki JE. Protocol for performing and optimizing differential scanning fluorimetry experiments. STAR Protoc 2023;4(4):102688. 10.1016/j.xpro.2023.102688. Dec 15. 37943662 PMC10663957

[30] Baljinnyam B, Ronzetti M, Yasgar A, Simeonov A. Applications of differential scanning fluorometry and related technologies in characterization of protein-ligand interactions. Methods Mol Biol 2020;2089:47–68. 10.1007/978-1-0716-0163-1_4. 31773647

[31] Niesen FH, Schultz L, Jadhav A, Bhatia C, Guo K, Maloney DJ, Pilka ES, Wang M, Oppermann U, Heightman TD, Simeonov A. High-affinity inhibitors of human NAD-dependent 15-hydroxyprostaglandin dehydrogenase: mechanisms of inhibition and structure-activity relationships. PLoS One 2010;5(11):e13719. 10.1371/journal.pone.0013719. Nov 2. 21072165 PMC2970562

[32] Langer A, Bartoschik T, Cehlar O, Duhr S, Baaske P, Streicher W. A new spectral shift-based method to characterize molecular interactions. Assay Drug Dev Technol 2022;20(2):83–94. 10.1089/adt.2021.133. Feb-Mar. 35171002 PMC8968852

[33] Golla K, Yasgar A, Manjuprasanna VN, Naik MU, Baljinnyam B, Zakharov AV, Jain S, Rai G, Jadhav A, Simeonov A, Naik UP. Small-molecule disruptors of the interaction between calcium- and integrin-binding protein 1 and integrin alpha (IIb)beta(3) as novel antiplatelet agents. ACS Pharmacol Transl Sci 2024;7(7):1971–82. 10.1021/acsptsci.4c00026. Jul 12. 39022362 PMC11249646

[34] Ronzetti MH, Baljinnyam B, Itkin Z, Jain S, Rai G, Zakharov AV, Pal U, Simeonov A. Application of temperature-responsive HIS-tag fluorophores to differential scanning fluorimetry screening of small molecule libraries. Front Pharmacol 2022;13:1040039. 10.3389/fphar.2022.1040039. 36506591 PMC9729254

[35] Zong G, Shears SB, Wang H. Structural and catalytic analyses of the InsP(6) kinase activities of higher plant ITPKs. FASEB J 2022;36(7):e22380. 10.1096/fj.202200393R. Jul. 35635723 PMC9202514

[36] Shears SB. Molecular basis for the integration of inositol phosphate signaling pathways via human ITPK1. Adv Enzyme Regul 2009;49(1):87–96. 10.1016/j.advenzreg.2008.12.008. 19200440 PMC4770455

[37] Jain S, Yasgar A, Dalal A, Davies M, Nilova A, Martinez N, Simeonov A, Rai G, Zakharov A. AI-driven drug discovery: identification and optimization of ALDH3A1 selective inhibitors with nanomolar activity. ChemRxiv 2024;2024(1101). 10.26434/chemrxiv-2024-5zxxm-v2.

[38] https://pubchem.ncbi.nlm.nih.gov/compound/219100.

[39] Porter K, Fairlie WD, Laczka O, Delebecque F, Wilkinson J. Idronoxil as an anticancer agent: activity and mechanisms. Curr Cancer Drug Tar 2020;20(5):341–54. 10.2174/1568009620666200102122830. 31899676

[40] https://pubchem.ncbi.nlm.nih.gov/compound/2877580.

[41] Wang H, Falck JR, Hall TM, Shears SB. Structural basis for an inositol pyrophosphate kinase surmounting phosphate crowding. Nat Chem Biol 2011;8(1):111–6. 10.1038/nchembio.733. Nov 27. 22119861 PMC3923263

[42] Imamura RM, Kumagai K, Nakano H, Okabe T, Nagano T, Kojima H. Inexpensive high-throughput screening of kinase inhibitors using one-step enzyme-coupled fluorescence assay for ADP detection. SLAS Discov 2019;24(3):284–94. 10.1177/2472555218810139. Mar. 30418800

[43] Yang X, Shears SB. Multitasking in signal transduction by a promiscuous human ins (3,4,5,6)P(4) 1-kinase/ins(1,3,4)P(3) 5/6-kinase. Biochem J 2000;351(3):551–5. Nov 1Pt 3(Pt. 11042108 PMC1221393

[44] Weaver JD, Wang H, Shears SB. The kinetic properties of a human PPIP5K reveal that its kinase activities are protected against the consequences of a deteriorating cellular bioenergetic environment. Biosci Rep 2013;33(2):e00022. 10.1042/BSR20120115. Feb 5. 23240582 PMC3564036

[45] Copeland RA. Evaluation of enzyme inhibitors in drug discovery. A guide for medicinal chemists and pharmacologists. Methods Biochem Anal 2005;46:1–265. 16350889

[46] Wu G, Yuan Y, Hodge CN. Determining appropriate substrate conversion for enzymatic assays in high-throughput screening. J Biomol Screen 2003;8(6):694–700. 10.1177/1087057103260050. Dec. 14711395

[47] Acker MG, Auld DS. Considerations for the design and reporting of enzyme assays in high-throughput screening applications. Perspectives in Science 2014;1(1–6):56–73.

[48] Haas JV, Eastwood BJ, Iversen PW, Devanarayan V, Weidner JR, Minimum significant ratio - A statistic to assess assay variability. In: Markossian S, Grossman A, Arkin M, et al., editors. Assay guidance manual; 2004. 24260775

[49] Auld DS, Thorne N, Boxer MB, Southall N, Shen M, Thomas CJ, Inglese J Understanding enzymes as reporters or targets in assays using quantitative high-throughput screening (qHTS). 2010:21–43.

[50] Wang Y, Jadhav A, Southal N, Huang R, Nguyen DT. A grid algorithm for high throughput fitting of dose-response curve data. Curr Chem Genomics 2010;4:57–66. 10.2174/1875397301004010057. Oct 21. 21331310 PMC3040458

[51] Huang R A quantitative high-throughput screening data analysis pipeline for activity profiling. Methods Mol Biol 2016;1473:111–22. 10.1007/978-1-4939-6346-1_12. 27518629

[52] Seethala R Handbook of drug screening, 13; 2009. p. 489 *(No Title)*.

[53] Fotsch C, Basu D, Case R, Chen Q, Koneru PC, Lo MC, Ngo R, Sharma P, Vaish A, Yi X, Zech SG, Hodder P. Creating a more strategic small molecule biophysical hit characterization workflow. SLAS Discov 2024;29(4):100159. 10.1016/j.slasd.2024.100159. Jun. 38723666

[54] Bergsdorf C, Wright SK. A guide to run affinity screens using differential scanning fluorimetry and surface plasmon resonance assays. Method Enzymol 2018;610:135–65. 10.1016/bs.mie.2018.09.015. 30390797

[55] Hawe A, Sutter M, Jiskoot W. Extrinsic fluorescent dyes as tools for protein characterization. Pharm Res-Dordr 2008;25(7):1487–99. 10.1007/s11095-007-9516-9. Jul. PMC2440933 18172579

[56] Scott AD Fluorescent thermal shift assays for identifying small molecule ligands. 2017.

[57] Liebschner D, Afonine PV, Baker ML, Bunkoczi G, Chen VB, Croll TI, Hintze B, Hung LW, Jain S, McCoy AJ, Moriarty NW, Oeffner RD, Poon BK, Prisant MG, Read RJ, Richardson JS, Richardson DC, Sammito MD, Sobolev OV, Stockwell DH, Terwilliger TC, Urzhumtsev AG, Videau LL, Williams CJ, Adams PD. Macromolecular structure determination using X-rays, neutrons and electrons: recent developments in Phenix. Acta Crystallogr D Struct Biol 2019;75(10):861–77. 10.1107/S2059798319011471. Oct 1Pt. 31588918 PMC6778852

[58] Emsley P, Cowtan K. Coot: model-building tools for molecular graphics. Acta Crystallogr D Biol Crystallogr 2004;60(12):2126–32. 10.1107/S0907444904019158. DecPtPt 1. 15572765

[59] Zakharov AV, Zhao T, Nguyen DT, Peryea T, Sheils T, Yasgar A, Huang R, Southall N, Simeonov A. Novel consensus architecture to improve performance of large-scale multitask deep learning QSAR models. J Chem Inf Model 2019;59(11):4613–24. 10.1021/acs.jcim.9b00526. Nov 25. 31584270 PMC8381874

[60] Wolber G, Langer T. LigandScout: 3-D pharmacophores derived from protein-bound ligands and their use as virtual screening filters. J Chem Inf Model 2005;45(1):160–9. 10.1021/ci049885e. Jan-Feb. 15667141

[61] Wolber G, Dornhofer AA, Langer T. Efficient overlay of small organic molecules using 3D pharmacophores. J Comput Aided Mol Des 2006;20(12):773–88. 10.1007/s10822-006-9078-7. Dec. 17051340

[62] Langer T, Wolber G. Pharmacophore definition and 3D searches. Drug Discov Today Technol 2004;1(3):203–7. 10.1016/j.ddtec.2004.11.015. Dec. 24981486

[63] Gu C, Wilson MS, Jessen HJ, Saiardi A, Shears SB. Inositol pyrophosphate profiling of two HCT116 cell lines uncovers variation in InsP8 levels. PLoS One 2016;11(10):e0165286. 10.1371/journal.pone.0165286. 27788189 PMC5082907

[64] Wilson MS, Bulley SJ, Pisani F, Irvine RF, Saiardi A. A novel method for the purification of inositol phosphates from biological samples reveals that no phytate is present in human plasma or urine. Open Biol 2015;5(3):150014. 10.1098/rsob.150014. Mar. 25808508 PMC4389793

[65] Qiu D, Wilson MS, Eisenbeis VB, Harmel RK, Riemer E, Haas TM, Wittwer C, Jork N, Gu C, Shears SB, Schaaf G, Kammerer B, Fiedler D, Saiardi A, Jessen HJ. Analysis of inositol phosphate metabolism by capillary electrophoresis electrospray ionization mass spectrometry. Nat Commun 2020;11(1):6035. 10.1038/s41467-020-19928-x. Nov 27. 33247133 PMC7695695

[66] Schrödinger release 2022–2. Schrödinger: LLC; 2021.

[67] Sastry GM, Adzhigirey M, Day T, Annabhimoju R, Sherman W. Protein and ligand preparation: parameters, protocols, and influence on virtual screening enrichments. J Comput Aided Mol Des 2013;27(3):221–34. 10.1007/s10822-013-9644-8. Mar. 23579614

[68] Prime. Schrödinger: LLC; 2025.

[69] Schrödinger release 2025–2: protein preparation workflow. Schrödinger: LLC; 2025.

[70] Epik. Schrödinger: LLC; 2024.

[71] Impact. Schrödinger, LLC.

[72] Schrödinger release 2015–1: ligprep. Schrödinger: LLC; 2015. Version 3.3.

[73] Friesner RA, Murphy RB, Repasky MP, Frye LL, Greenwood JR, Halgren TA, Sanschagrin PC, Mainz DT. Extra precision glide: docking and scoring incorporating a model of hydrophobic enclosure for protein-ligand complexes. J Med Chem 2006;49(21):6177–96. 10.1021/jm051256o. Oct 19. 17034125

[74] Halgren TA, Murphy RB, Friesner RA, Beard HS, Frye LL, Pollard WT, Banks JL. Glide: a new approach for rapid, accurate docking and scoring. 2. Enrichment factors in database screening. J Med Chem 2004;47(7):1750–9. 10.1021/jm030644s. Mar 25. 15027866

[75] Southall NT, Jadhav A, Huang R, Nguyen T, Wang Y. Enabling the large-scale analysis of quantitative high-throughput screening data. Handbook of drug screening. CRC Press; 2016. p. 456–78.

