# Supplementary material for: A Hybrid Experimental and in silico Platform for ITPK1 Chemical Probe Discovery
Source: SLAS Discov. Author manuscript; Available in PMC 2026 Jul 24. (PMC13397342; doi:10.1016/j.slasd.2026.100323)
Supplement: mmc1 [file NIHMS2196165-supplement-mmc1.zip › Supplementary Information_Material and Methods_v1.docx]

**Supplementary Information**

**A Platform for Inositol-tetrakisphosphate 1-kinase (ITPK1) Inhibitor Discovery: An Integrated Machine Learning and High-Throughput Screening Approach**

Adam Yasgar*^1*^*, Sankalp Jain*^1^*, Huanchen Wang^2^, Guangning Zong*^2^*, Stephen Shears*^2^*, Robin E. Stanley*^2^*, Chih-Shia Lee*^3^*, Haibo Zhang*^3^*, Ji Luo*^3^*, Eric Lindberg*^4^*, Carolyn Woodroofe*^4^*, Kelly Lane*^4^*, Burchelle Blackman*^4^*, Dan Crook*^4^*, Hsiuling Lin*^1^*, Bolormaa Baljinnyam*^1^*, Michael Ronzetti*^1^*, Anton Simeonov*^1^*, Sandeep Rana*^1^*, Ganesha Rai*^1^*, Alexey V. Zakharov*^1^* and Natalia J. Martinez*^1^*^*^

*^1^*National Center for Advancing Translational Sciences, National Institutes of Health, Rockville, MD, United States of America.

*^2^* Molecular and Cellular Biology Laboratory, National Institute of Environmental Health Sciences, National Institutes of Health, Research Triangle Park, North Carolina, USA.

*^3^*Laboratory of Cancer Biology and Genetics, Center for Cancer Research, National Cancer Institute, National Institutes of Health Bethesda, MD 20892

*^4^*Chemistry and Synthesis Center, National Heart, Lung, and Blood Institute, Bethesda, MD 20814

*Corresponding author: Adam Yasgar and Natalia Martinez

**Table of Contents**

Experimental for Synthesis of Inositol Phosphates Page S-3

**Materials and Methods**

**Experimental for Synthesis of Inositol Phosphates**

**General Methods**. Chemicals and reagents: All materials were purchased from commercial sources and used without further purification unless otherwise noted. Deuterated solvents were purchased from Cambridge Isotope Labs. Phytic acid sodium salt hydrate (InsP_6_) was used without further purification. Prior to reaction, the InsP_6_ was dissolved in water and the pH was adjusted to 6.5 with HCl. The stock concentration was 37 mM based on the free acid molecular weight (647.9 g/mol) and 70% purity based on NMR. A stock solution of ATP was prepared in water, the pH was adjusted to 6-7 with NaOH and the concentration was determined using ε_259_= 15.4 mM^-1^cm^-1^. Creatine Kinase (CK) was prepared at 350 U/mL in 200 mM MOPS pH 6.5 buffer, containing 20 mM MgCl_2_ and 20 mM DTT. His-EhI6KA enzyme (32.7 kDa) and PPIP5K2^KD^ enzyme (Sumo-HsIP7K2; 55.3 kDa) were both supplied in 20 mM Tris-HCl, 150 mM NaCl, pH 7.2. Amberlite Resin (Alfa Aesar™ Amberlite™ IRC-748, ion exchange resin) was pre-equilibrated as follows. 25-50 mL was placed on a fritted filter and washed with 100 mL MeOH followed by 500-1000 mL water. The resin was then acidified with 250-500 mL of 1M HCl. Next the resin was washed with water until neutral pH was reached. Lastly, the resin was equilibrated with 250-500 mL 1 M ammonium bicarbonate and washed with 1.0 L water.

**Instrumentation**. NMR spectra were recorded on Varian spectrometers operating at 400 MHz for proton nuclei, 100 MHz for carbon nuclei or 162 MHz for phosphorous nuclei. Concentration and purity of D_2_O solutions of InsP_x_ was determined by NMR against a standard (phosphonoacetic acid). High-resolution mass spectrometry was performed by direct inject on a Xevo G2-XS QTof Quadrupole Time-of-Flight Mass Spectrometry (Waters), using negative ion mode. Ion Chromatography-Mass Spectrometry (IC-MS) was kindly performed by Dan Crooks (NCI) on a Thermo-Scientific Orbitrap Fusion Lumos equipped with a Thermo-Scientific ICS-6000, using anion suppressed conductivity.

**3,4,5,6-InsP_4_**

1d-*myo*-Inositol 3,4,5,6-tetrakisphosphate was synthesized loosely following the protocol previously described by Mills et al^1^.

**d-1,2-Di-O-benzyl-3,4,5,6-tetrakis[di(benzyloxy)phospho]-myo-inositol.** To an oven-dried flask under Ar were added d-1,2-Di-O-benzyl-myo-inositol (73 mg, 203 umol), 5-phenyltetrazole (257 mg, 1.76 mmol, 8.67 equiv) and 0.5 mL DCM. The stirred suspension was treated with bis(benzyloxy)diisopropylaminophosphine (350 mg, 340 uL, 1.01 mmol, 5 equiv) and the reaction was monitored by ^31^P NMR. After 2 h at RT, the reaction was cooled to -78 °C, diluted with 3 mL dry DCM and treated with mCPBA (280 mg, 1.62 mmol, 8 equiv). The reaction was allowed to stir overnight with warming to RT. Saturated aqueous NaHCO_3_ (40 mL) was added and the product was extracted with 3 x 40 mL DCM. The organic layers were dried over Na_2_SO_4_ and concentrated under reduced pressure. The product (270 mg, 19.2 umol, 95% yield) was isolated by preparative HPLC eluting with a gradient from 70->100% MeCN in 0.05% aqueous TFA. Characterization was consistent with previous reports.

**d-*myo*-Inositol 3,4,5,6-tetrakisphosphate (3,4,5,6-InsP_4_).** d-1,2-Di-O-benzyl-3,4,5,6-tetrakis[di(benzyloxy)phospho]-myo-inositol (269 mg, 19.2 umol) was stirred in 5:1 methanol: water (12 mL) under a hydrogen balloon for 4 days and monitored by LCMS. Upon completion, the reaction was flushed with Ar and filtered through Celite. Solvents were removed under reduced pressure and the resulting residue was redissolved in water and subjected to preparative HPLC eluting with 100% 10 mM aqueous NH_4_OAc for 4 min followed by a gradient to 25% MeCN over 6 min. Product-containing fractions were identified by LCMS, combined, and lyophilized to yield 94.3 mg of a white solid. 89 mg of this solid were taken up in 5 mL of water and 2 mL of 2% aqueous formic acid and loaded in equal portions onto five Oasis WAX columns (150 mg) that had been preconditioned with successive washes with 7 mL each of MeOH, H_2_O, 50 mM aqueous NH_4_CO_2_H, and H_2_O. The columns were washed with 1.4 mL H2O, then MeOH and H2O, and finally eluted with 7:2:1 H2O:MeOH:aqueous NH4OH. Fractions were analyzed by LCMS and those containing product were concentrated under reduced pressure and then lyophilized to yield 71 mg of the product as a white solid, containing 0.3 molar equiv of formic acid. Characterization was consistent with previous reports.

**1,3,4,5,6-InsP5**

*myo*-inositol 1,3,4,5,6 pentakisphosphate was synthesized following the protocol previously described by Godage *et al*. with a few modifications^2^.

**2-O-Benzoyl 1,3,4,5,6-Pentakis-O-[bis(benzyloxy)-phosphoryl]-*myo*-Inositol.** To an oven dried flask under argon was added 2-O-benzoyl *myo*-inositol (1 g, 3.52 mmol, 1 equiv) and 5-phenyltetrazole (5.3 g, 35.2 mmol, 10 equiv) and 10 mL of dry dichloromethane. Bis(benzyloxy)(*N,N*-diisopropylamino) phosphosphine (9.32 mL, 25.0 mmol, 7.1 equiv) was added dropwise and the reaction was stirred at room temperature for 2 hours. The reaction mixture was then cooled to -40 deg C and 77% *m*-CPBA (7.88 g, 35.2 mmol, 10 equiv) was added. The reaction left to reach room temperature and after 30 minutes the reaction mixture was diluted with dichloromethane and washed with 10% sodium sulfite, brine, dried over sodium sulfate, filtered, and concentrated *in vacuo*. The residue was purified by flash chromatography (chloroform/acetone, 0 to 15% acetone) to yield the final product (4.04 g, 72%) as a colorless oil. Characterization was consistent with previous reports.

***myo*-Inositol, 2-benzoate 1,3,4,5,6-pentakis(dihydrogen phosphate).** To a three-necked flask under argon was added palladium hydroxide on carbon (10%)(460 mg). The catalyst was added as a solid and washed down with water (10 mL). A solution of 2-O-Benzoyl 1,3,4,5,6-Pentakis-O-[bis(benzyloxy)-phosphoryl]-*myo*-Inositol (4.04 g, 2.55 mmol) in methanol (15 mL) was added to the flask, followed by an additional 95 mL of Methanol. The flask was swiftly evacuated under vacuum and flushed with argon, and this was repeated a total of three times. A balloon of hydrogen gas was attached and the flask was evacuated under vacuum and flushed with hydrogen gas, and this was repeated three times. The reaction was left to stir at room temperature overnight. The reaction mixture was then filtered through a pad of celite and concentrated under reduced pressure to yield the product in quantitative yield (1.74 g, 2.54 mmol). Characterization was consistent with previous reports.

***myo*-inositol 1,3,4,5,6 pentakisphosphate.** To pyrex flask was added *myo*-Inositol, 2-benzoate 1,3,4,5,6-pentakis(dihydrogen phosphate) (310 mg, 0.45 mmol) was added 15 mL aqueous ammonia (28-30%) at heated at 60 deg C overnight. The solution was then concentrated under reduced pressure. The original protocol called for washing with chloroform to remove the benzamide byproduct, however we found that a significant amount of benzamide remained, so we modified the protocol. The concentrated residue was suspended in methanol (40 mL) and centrifuged (233 x g) for 10 minutes at 4 deg C, discarding the supernatant. This was repeated three times. The white solid was then suspended in chloroform and centrifuged for 10 minutes at 10 deg C. This was also repeated thrice. The white solid was then dissolved in water and lyophilized to yield the desired product in its hexaammonium salt form (250 mg, 0.37 mmol, 81%). Characterization was consistent with previous reports.

**5PP-InsP_5_ and 1,5(PP)_2_-InsP_4_**

Both 5-InsP_7_ and 1,5-InsP_8_ were synthesized following the protocol published by Puschmann *et al*^3^.

**5-InsP_7_ Enzymatic Synthesis.** InsP_6_ (175 mg; 0.27 mmol), 2 mM ATP, 5 mM creatine phosphate (CP), 7 mM MgCl_2_, and 1 mM DTT were added to pre-warmed 20 mM MES pH 6.4, 50 mM NaCl buffer at a total volume of 1.08 L. The mixture was divided evenly between two 1.0 L bottles and was incubated in a water bath at 37°C for 10 minutes. His-EhIP6KA (to 0.3 µM) and CK (to 1 U/mL) were then added. The bottles were gently mixed and kept at 37°C for 30 minutes. The reaction mixture was then quickly cooled to 4°C using a dry ice-isopropanol bath. 10.5 g of C_18_ reversed phase silica gel was packed onto a fritted filter and washed with 50 mL acetonitrile followed by 50 mL water. The reaction mixture was then passed through the filter under vacuum, followed by 2 x 50 mL water, and all eluents were combined. 50 equivalents of MgCl_2_ (over InsP_6_ starting concentration) were added and the pH was adjusted to 8.8 with NaOH. The mixture was left overnight at room temperature to allow 5-InsP_7_ to precipitate as the magnesium complex. The suspension was collected in 50 mL canonical centrifuge tubes and centrifuged at 3000 x g for 2 minutes. The supernatant was removed and the precipitate was combined into 2 tubes and washed 3 times with 15 mL 8 mM MgCl_2_ pH 8.9. Equal volumes (20 mL) of 10 mM ammonium bicarbonate and pre-equilibrated Amberlite IRC-748 resin were added to each tube and vortexed until the precipitate dissolved. The suspension was added to 10 mL pre-equilibrated Amberlite IRC-748 on a fritted filter and the solubilized product was separated and collected under vacuum. The resin was washed with water and all eluents were combined and lyophilized to yield the product as the ammonium salt (173 mg; 0.24 mmol, 89% yield based on free acid molecular weight of 726.9 g/mol; >80% purity).

**1,5-InsP_8_ Enzymatic Synthesis.** The 5-InsP_7_ starting material was synthesized following the protocol above and dissolved in water immediately prior to reaction. The purity of 86.8% (as determined by IC-MS) was considered when determining the stock concentration. 5-InsP_7_ (50 mg; 69 µmol), 2 mM ATP, 5 mM CP, 5 mM MgCl_2_, and 1 mM DTT were added to pre-warmed 20 mM MES pH 6.4, 250 mM NaCl buffer to give a total volume of 357.5 mL. The mixture was incubated in a water bath at 37°C for 10 minutes. PPIP5K2^KD^ (to 1.5 µM) and CK (to 1 U/mL) were then added. The bottles were gently mixed and left to react at 37°C for 5.5 hours. The reaction mixture was then quickly cooled to 4°C using a dry ice-isopropanol bath. C_18_ reversed phase silica gel, 6.0 g, was packed onto a fritted filter and washed with 30 mL acetonitrile followed by 30 mL water. The reaction mixture was then passed through the filter under vacuum, followed by 2 x 30 mL water, and all eluents were combined. 50 equivalents of MgCl_2_ (over 5-InsP_7_ starting concentration) were added and the pH was adjusted to 8.8 with NaOH. The mixture was left overnight at room temperature to allow 1,5-InsP_8_ to precipitate as the magnesium complex. The suspension was collected in 50 mL canonical tubes and centrifuged at 3000 x g for 2 minutes. The supernatant was removed and the precipitate was combined into one tube and washed 3 times with 15 mL 8 mM MgCl_2_ pH 8.9. Equal volumes (15 mL) of 10 mM aqueous ammonium bicarbonate and pre-equilibrated Amberlite IRC-748 resin were added to the tube and the mixture was vortexed until the precipitate dissolved. The suspension was added to 10 mL pre-equilibrated Amberlite IRC-748 on a fritted filter and the solubilized product was separated and collected under vacuum. The resin was washed with water and all eluents were combined and lyophilized to yield the product as the ammonium salt (47 mg; 58 µmol, 84% yield based on free acid molecular weight of 805.9 g/mol; purity 71% by IC-MS).

**References**

1. Mills SJ, Riley AM, Liu C, Mahon MF, Potter BV. A definitive synthesis of D-myo-inositol 1,4,5,6-tetrakisphosphate and its enantiomer D-myo-inositol 3,4,5,6-tetrakisphosphate from a novel butane-2,3-diacetal-protected inositol. *Chemistry*. Dec 15 2003;9(24):6207-14. doi:10.1002/chem.200305207

2. Godage HY, Riley AM, Woodman TJ, Thomas MP, Mahon MF, Potter BV. Regioselective opening of myo-inositol orthoesters: mechanism and synthetic utility. *J Org Chem*. Mar 15 2013;78(6):2275-88. doi:10.1021/jo3027774

3. Puschmann R, Harmel RK, Fiedler D. Scalable Chemoenzymatic Synthesis of Inositol Pyrophosphates. *Biochemistry*. Sep 24 2019;58(38):3927-3932. doi:10.1021/acs.biochem.9b00587
