# Supplementary material for: A Hybrid Experimental and in silico Platform for ITPK1 Chemical Probe Discovery
Source: SLAS Discov. Author manuscript; Available in PMC 2026 Jul 24. (PMC13397342; doi:10.1016/j.slasd.2026.100323)
Supplement: mmc2 [file NIHMS2196165-supplement-mmc2.zip › ITPK1 HTS Assay Paper Figures_Supplemental_v8.pptx]

## Slide 1
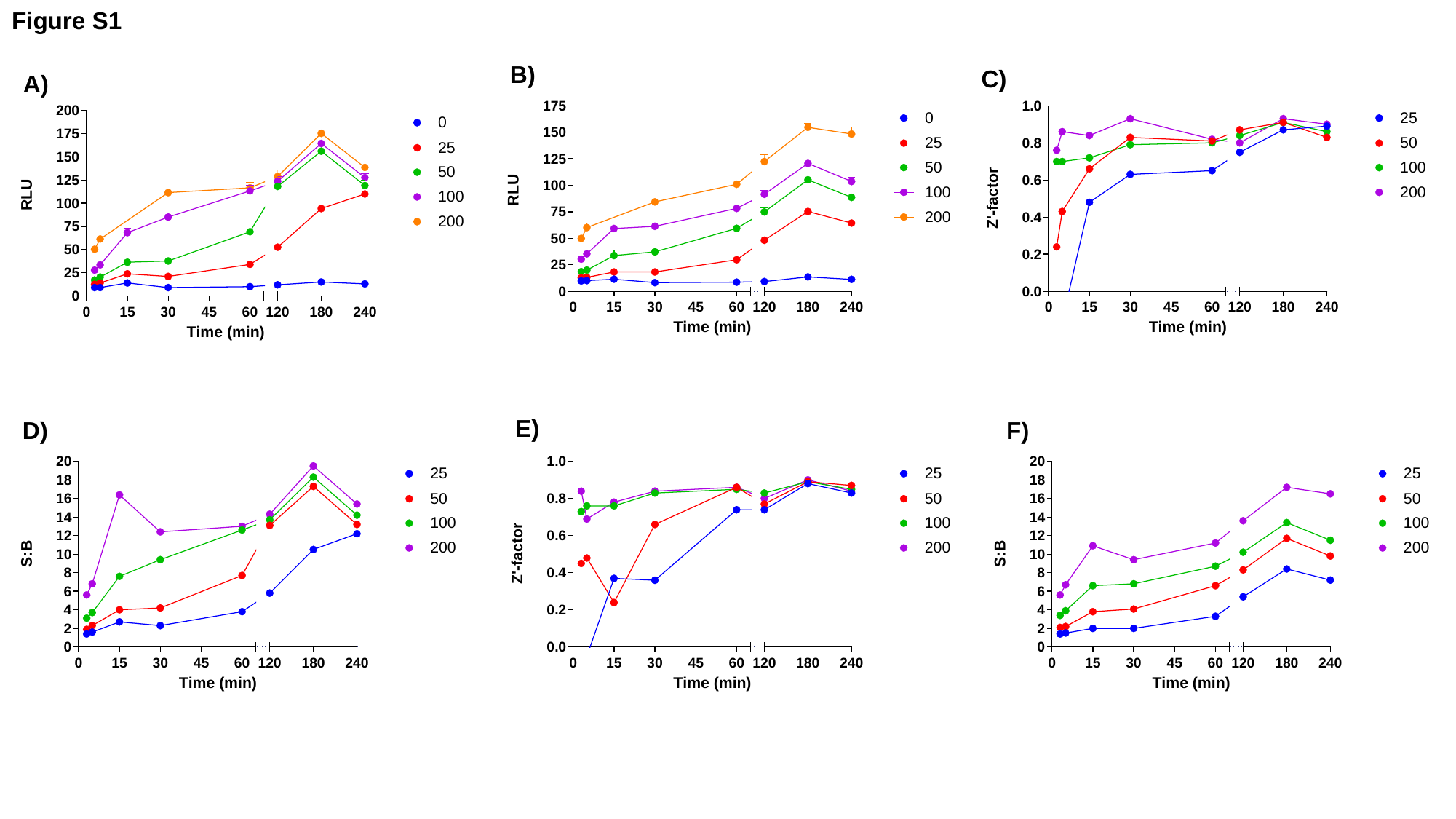

Figure S1
B)
C)
A)
E)
D)
F)

## Slide 2
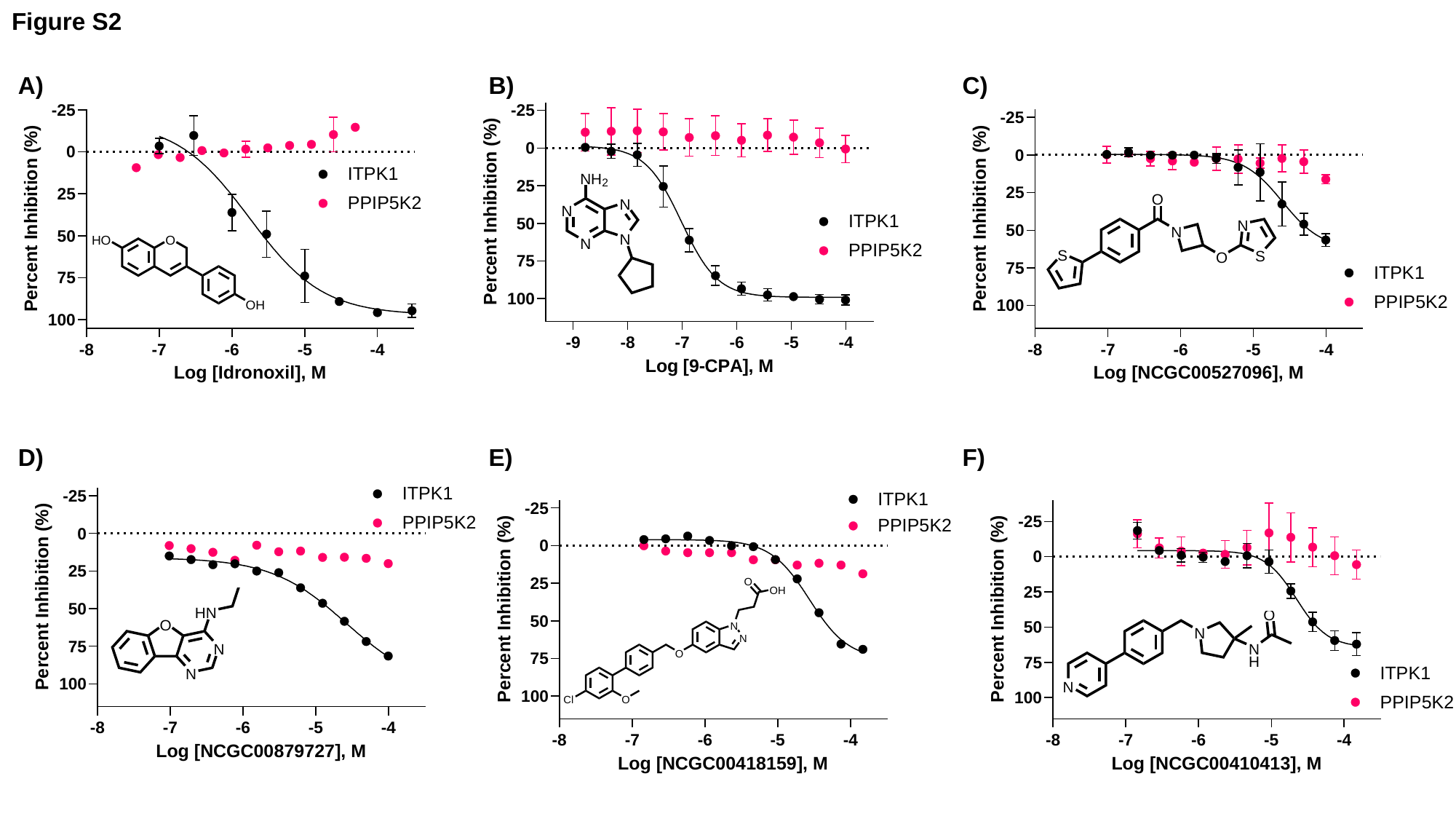

Figure S2
A)
B)
C)
D)
E)
F)

## Slide 3
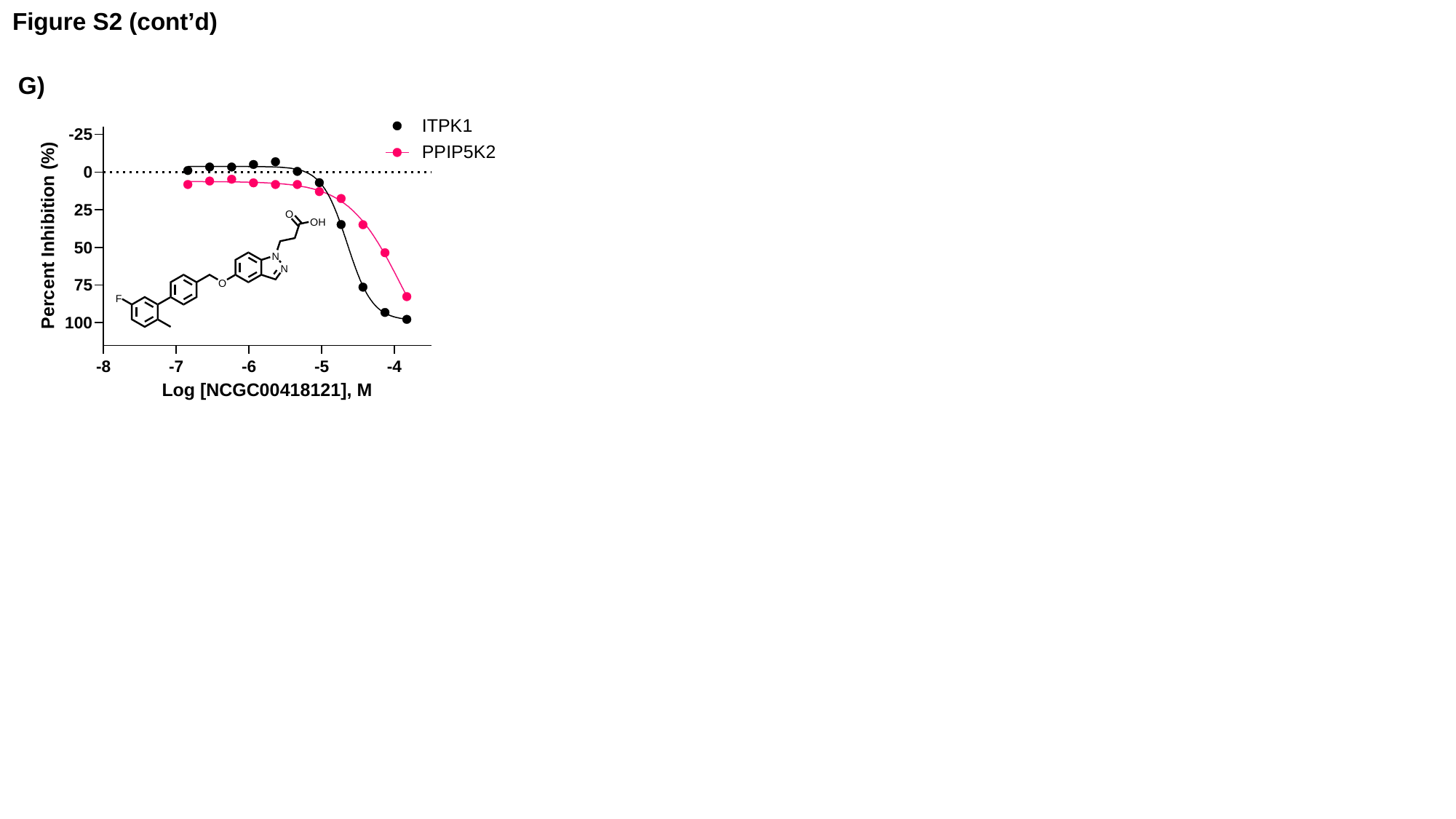

Figure S2 (cont’d)
G)

## Slide 4
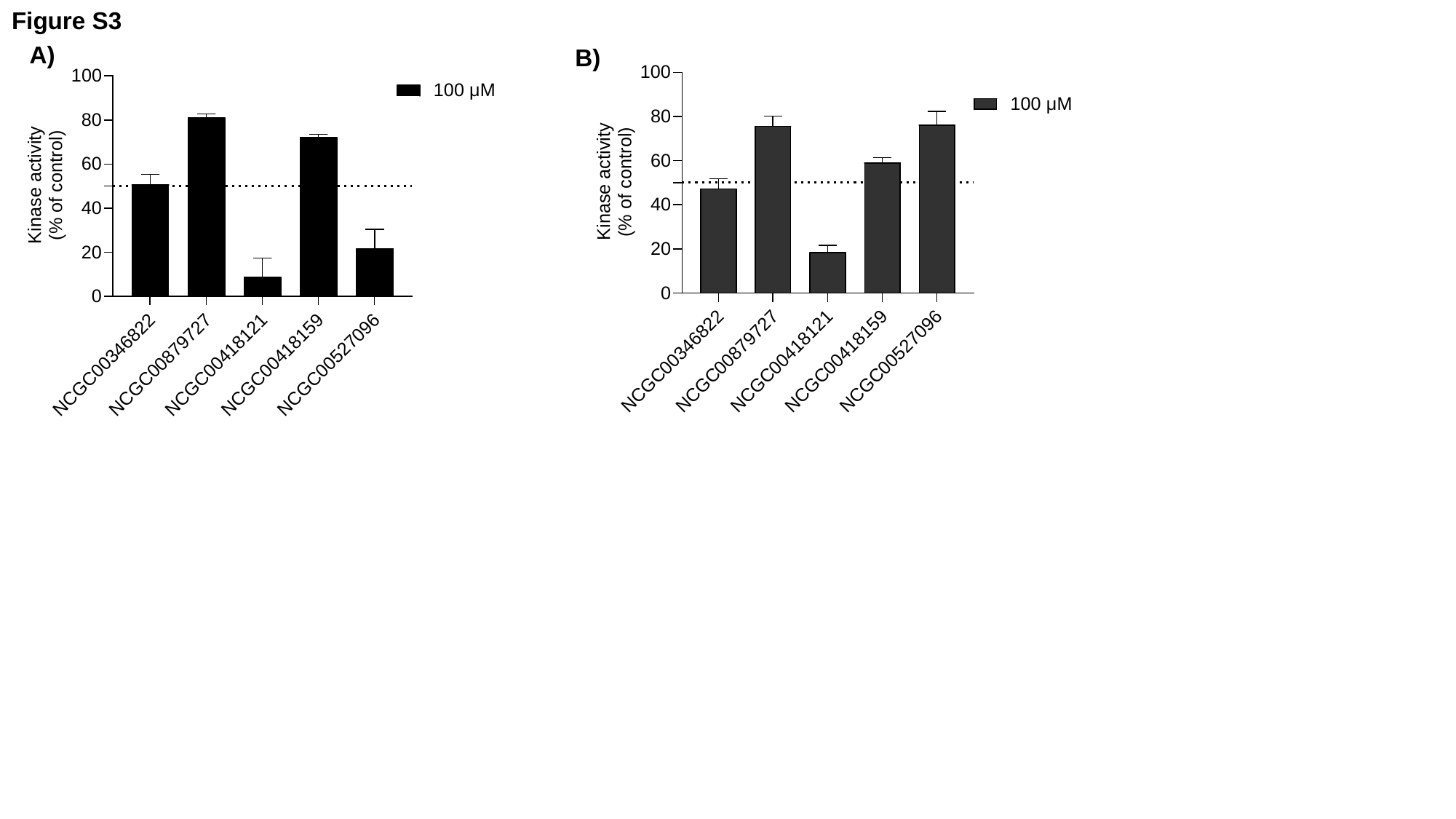

Figure S3
A)
B)

## Slide 5
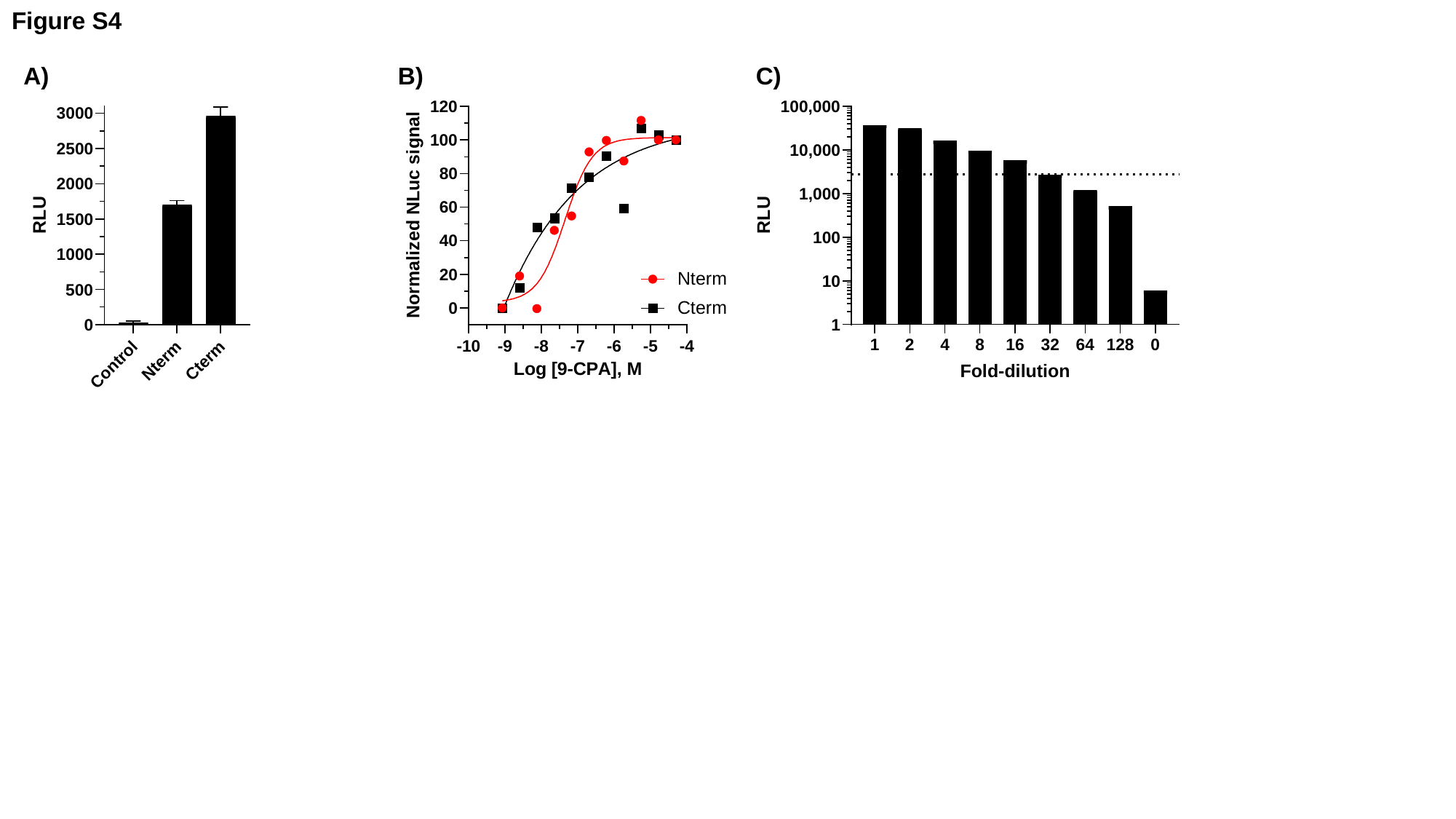

Figure S4
A)
B)
C)

## Slide 6
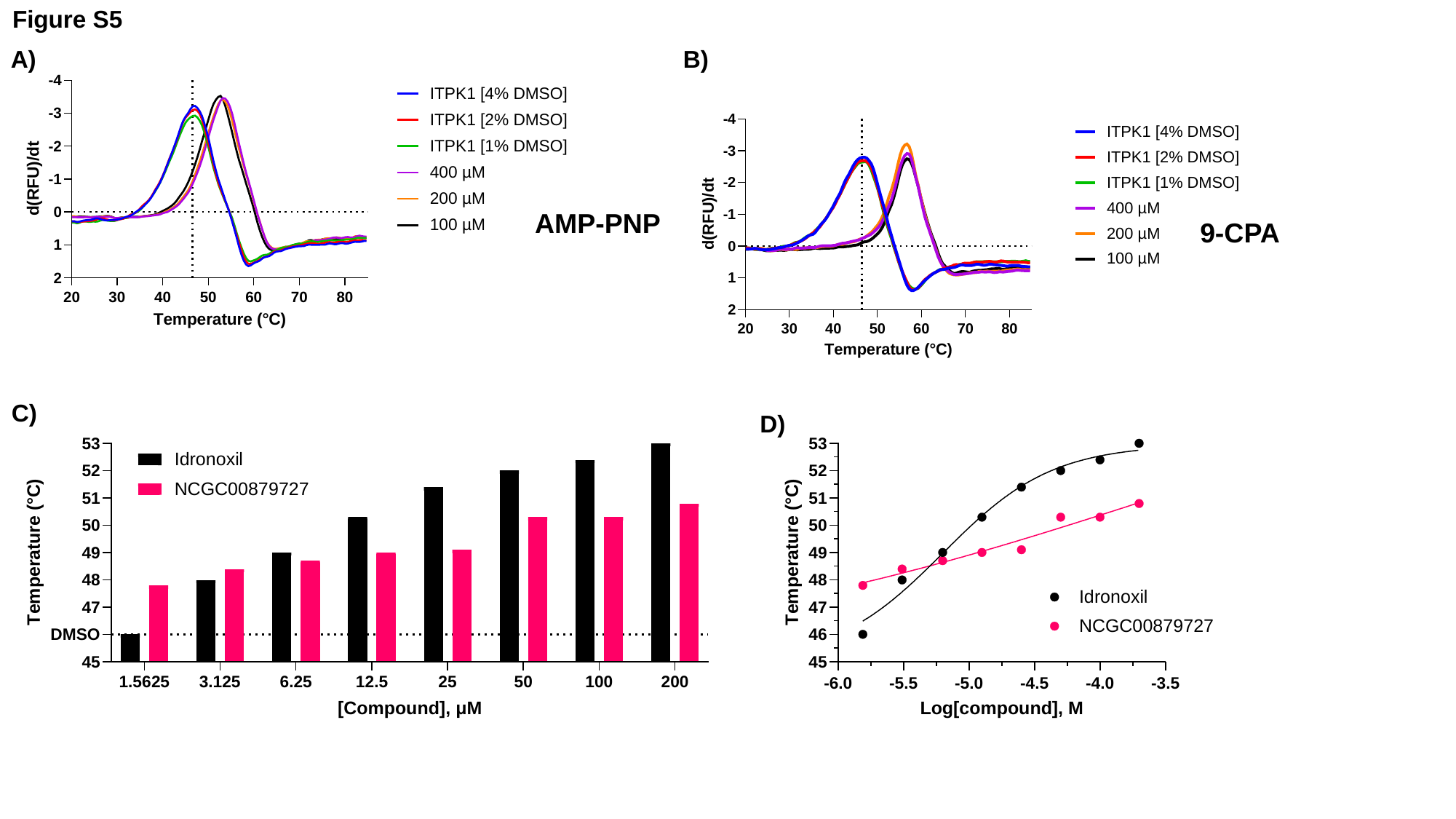

Figure S5
A)
B)
AMP-PNP
9-CPA
C)
D)

## Slide 7
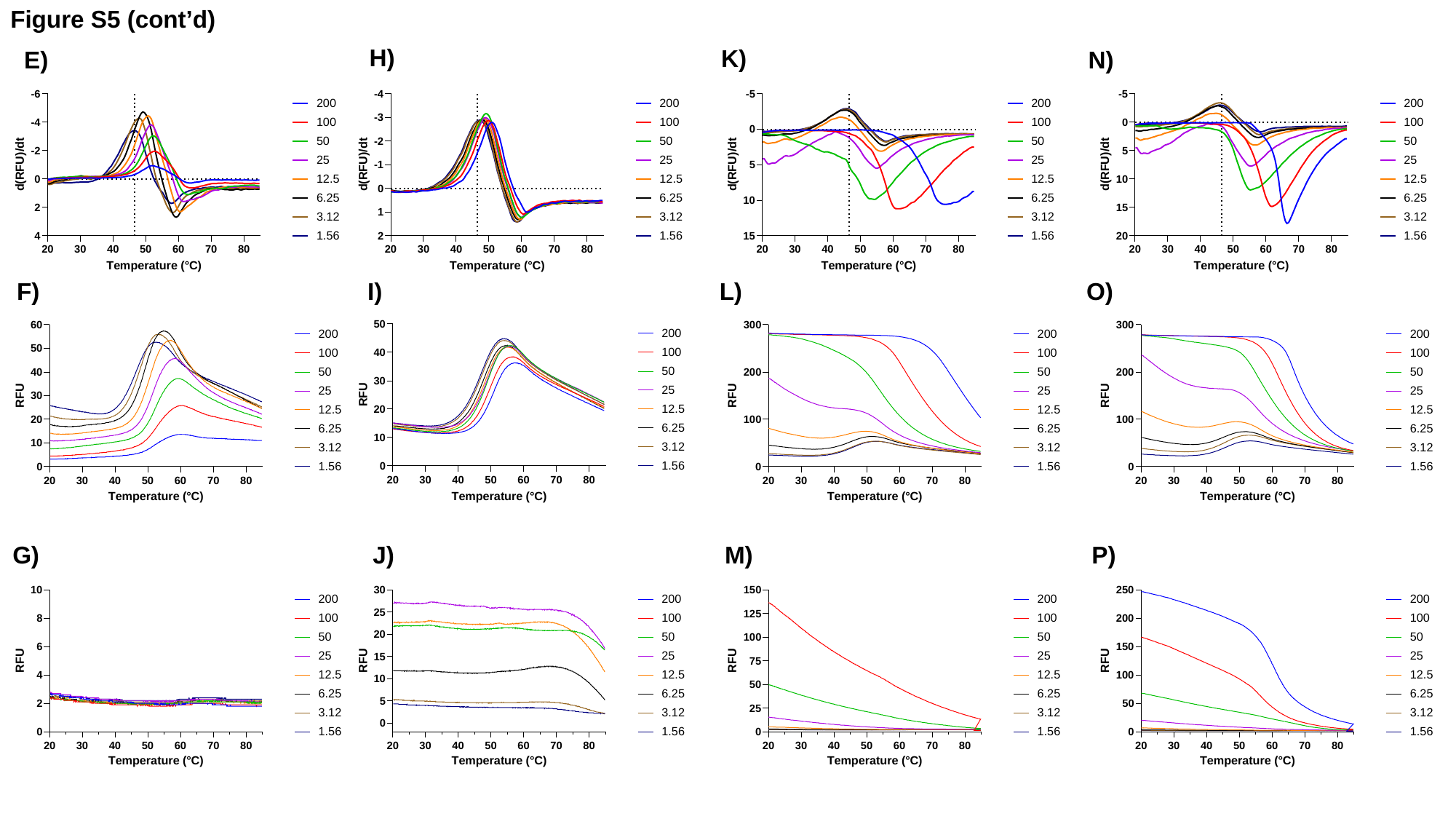

Figure S5 (cont’d)
H)
K)
E)
N)
F)
I)
L)
O)
G)
J)
M)
P)

## Slide 8
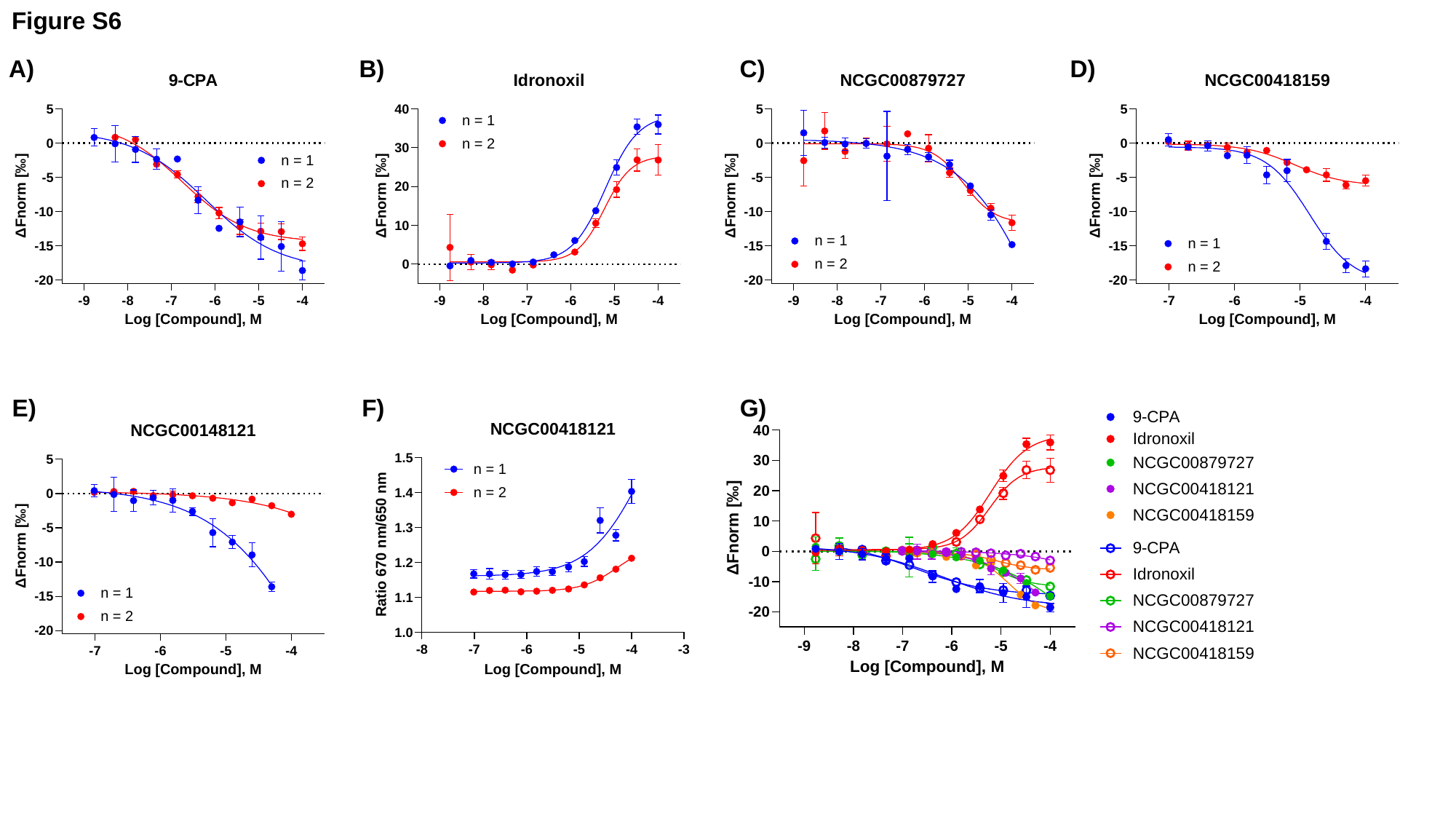

Figure S6
A)
B)
C)
D)
E)
F)
G)

## Slide 9
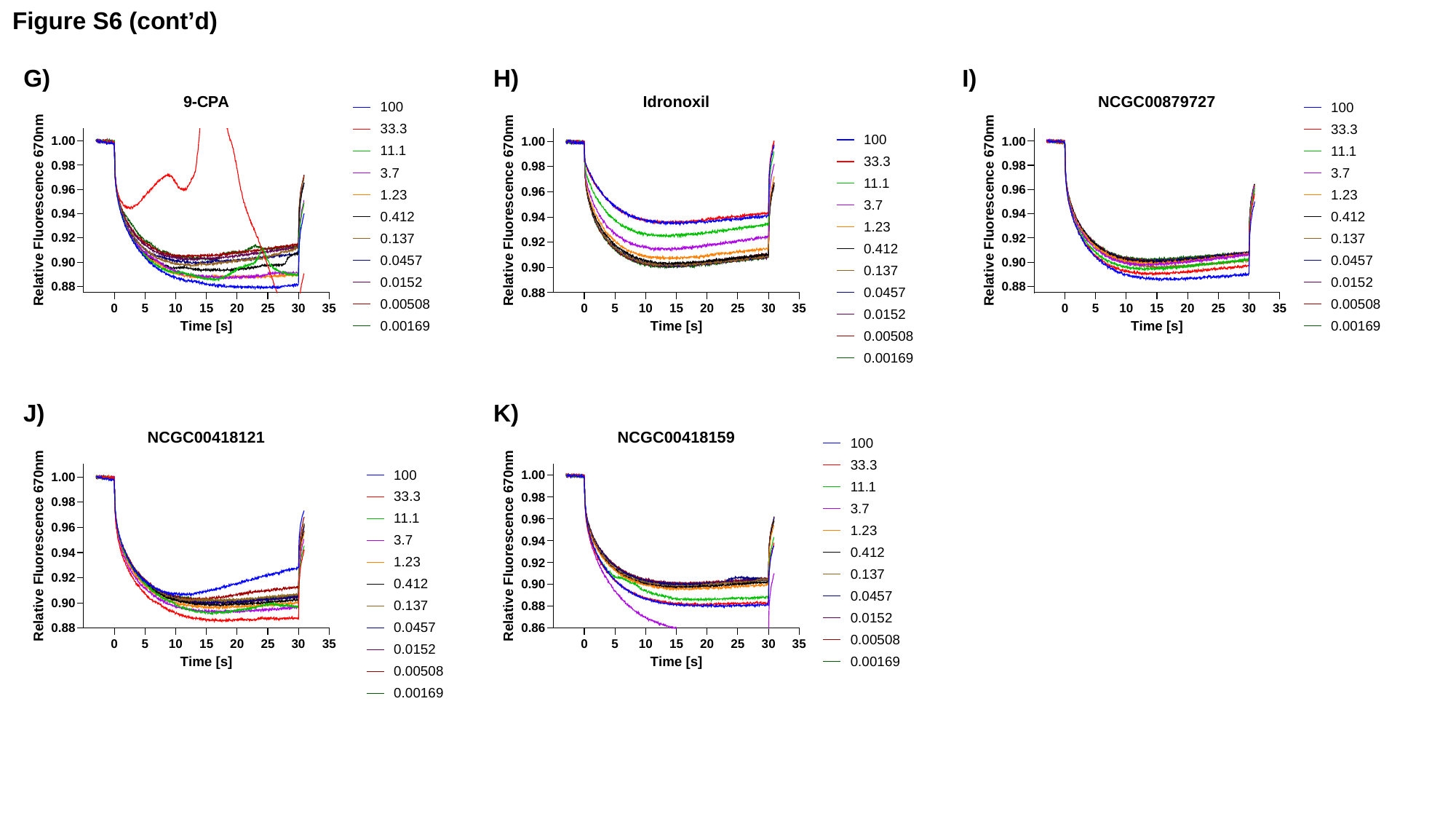

Figure S6 (cont’d)
G)
H)
I)
J)
K)

## Slide 10
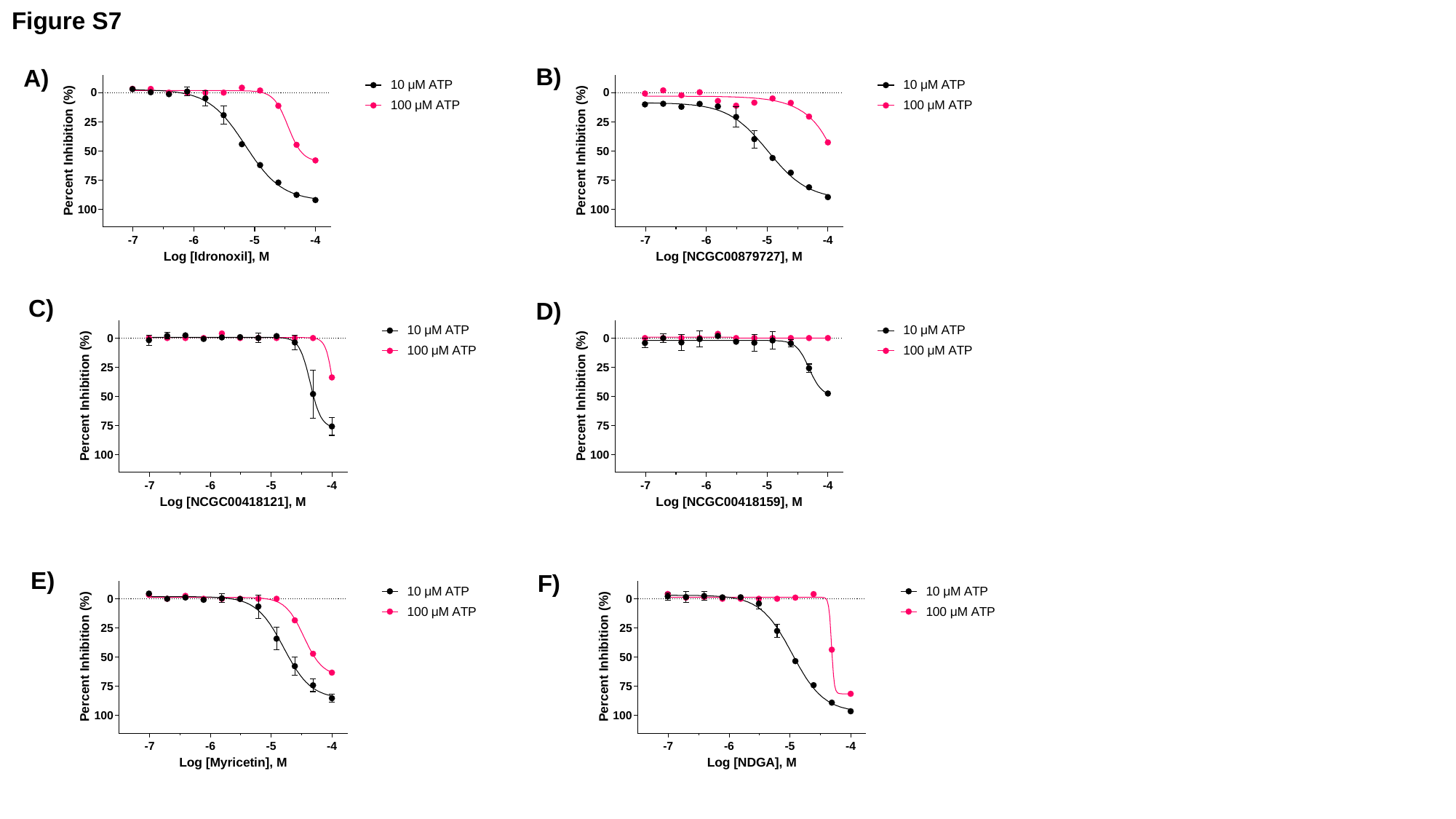

Figure S7
B)
A)
C)
D)
E)
F)

## Slide 11
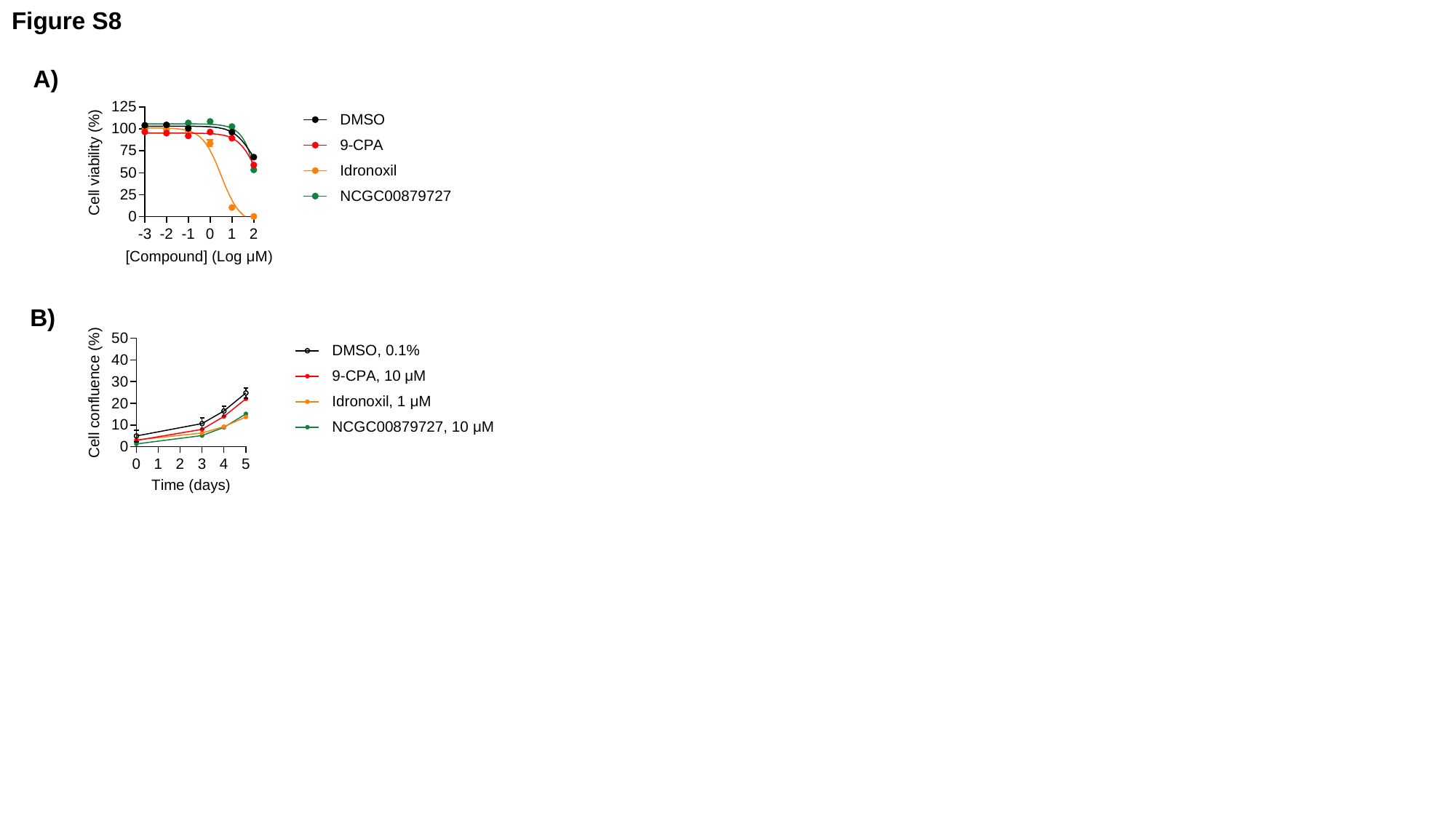

Figure S8
A)
B)

## Slide 12
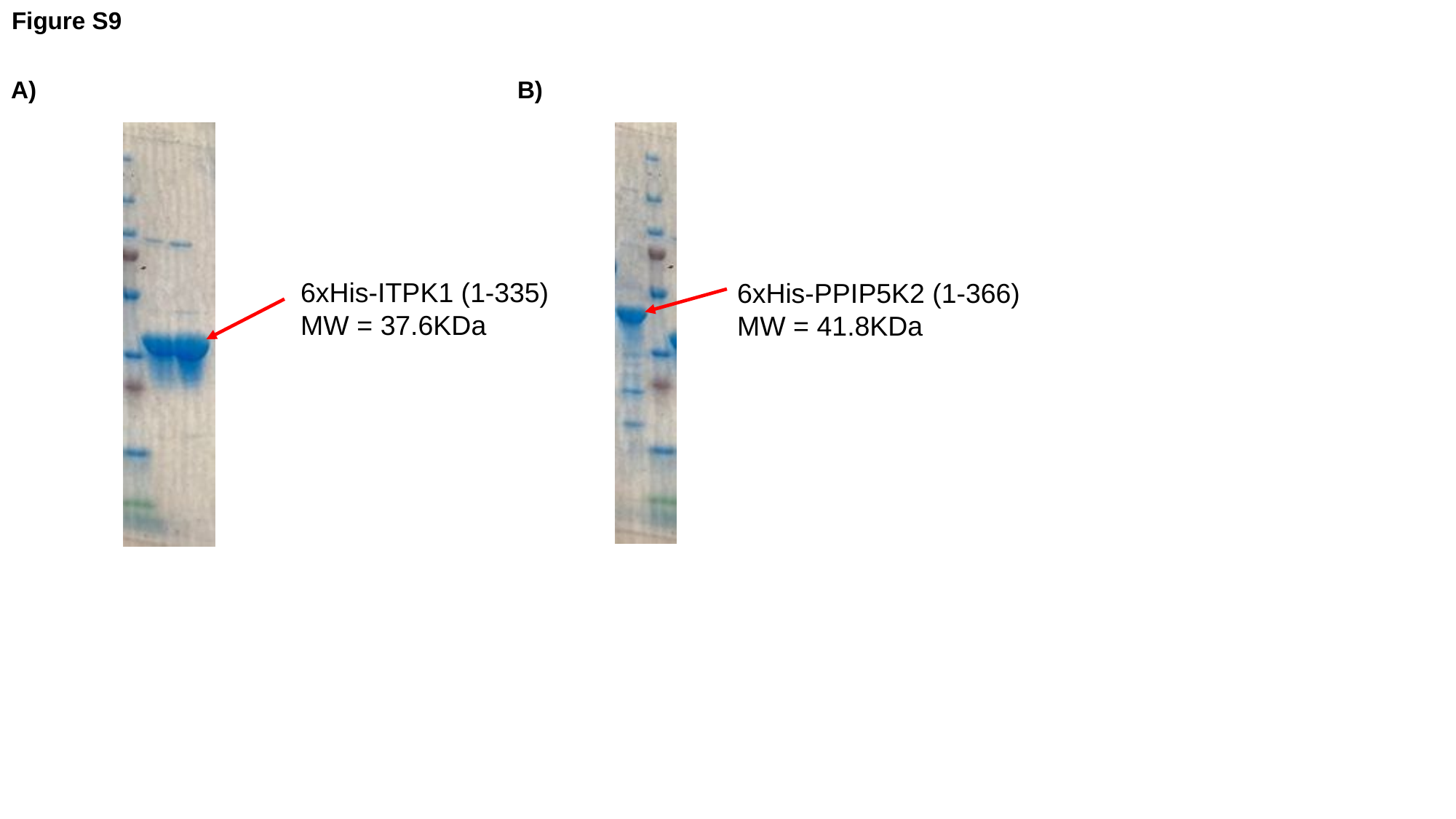

Figure S9
A)
B)
6xHis-ITPK1 (1-335)
MW = 37.6KDa
6xHis-PPIP5K2 (1-366)
MW = 41.8KDa

## Slide 13
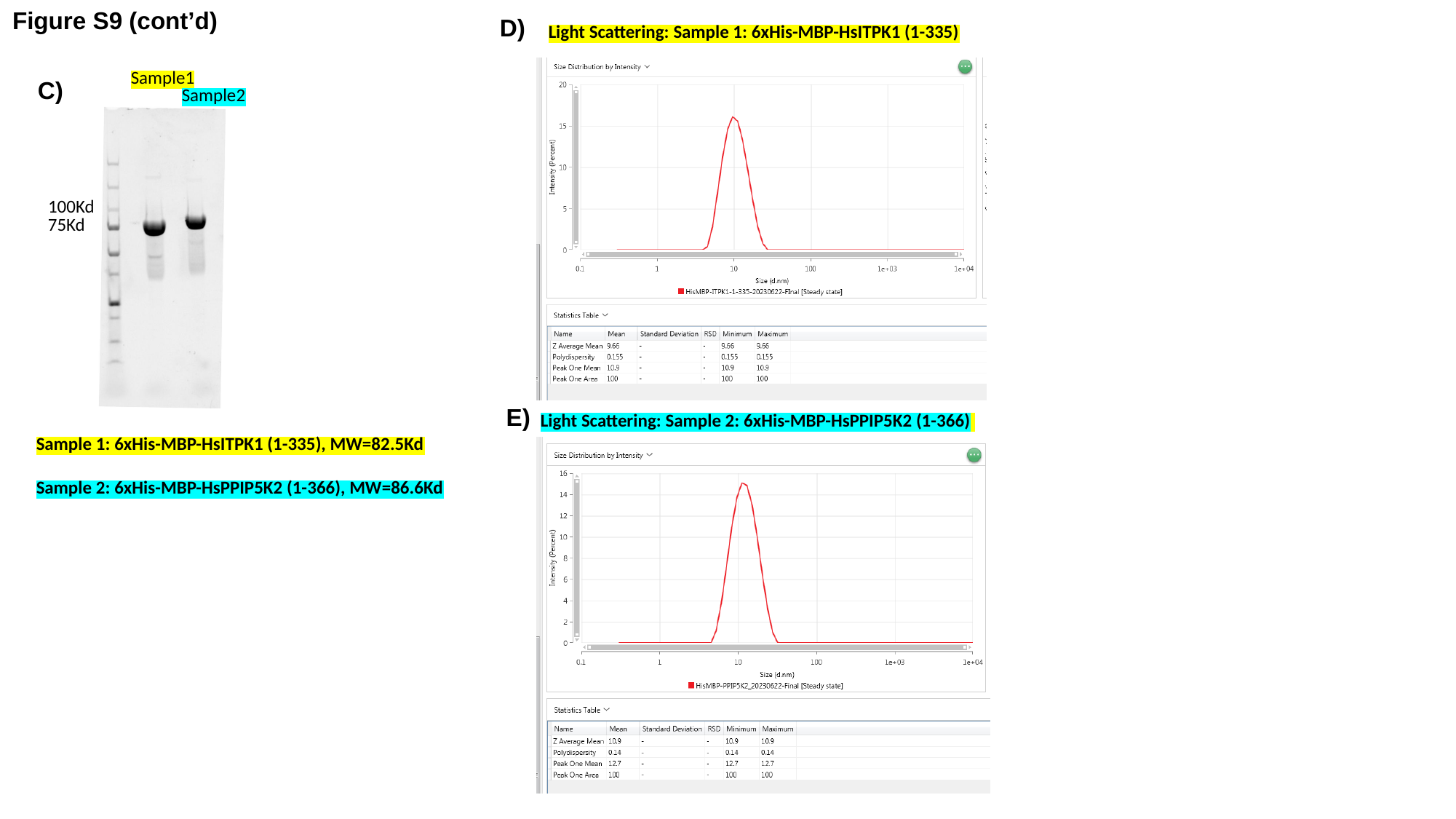

Figure S9 (cont’d)
D)
Light Scattering: Sample 1: 6xHis-MBP-HsITPK1 (1-335)
Sample1
C)
Sample2
100Kd
75Kd
E)
Light Scattering: Sample 2: 6xHis-MBP-HsPPIP5K2 (1-366)
Sample 1: 6xHis-MBP-HsITPK1 (1-335), MW=82.5Kd
Sample 2: 6xHis-MBP-HsPPIP5K2 (1-366), MW=86.6Kd

## Slide 14
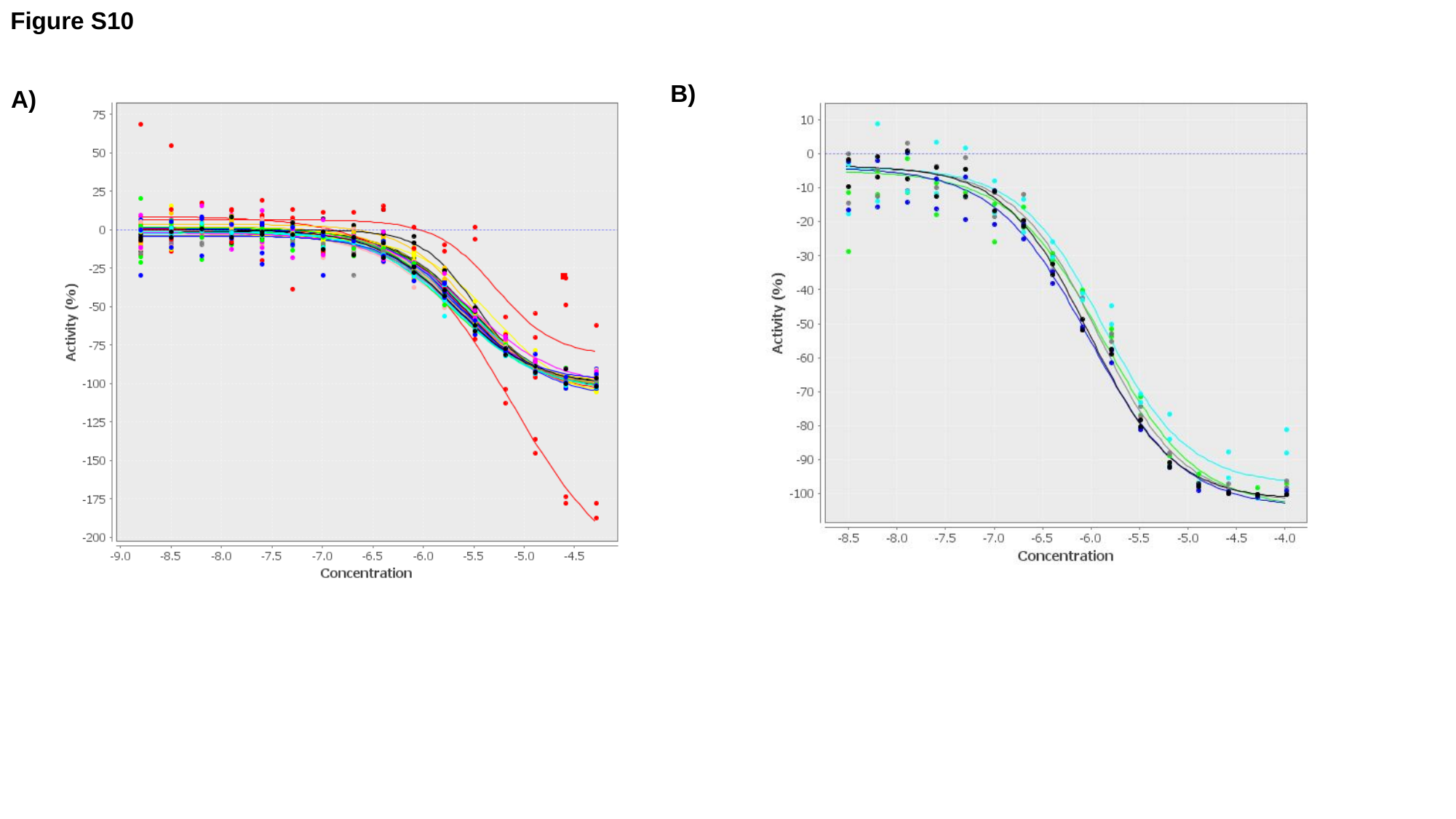

Figure S10
B)
A)
